# Preconception care: screening and management of chronic disease and promoting psychological health

**DOI:** 10.1186/1742-4755-11-S3-S5

**Published:** 2014-09-26

**Authors:** Zohra S Lassi, Ayesha M Imam, Sohni V Dean, Zulfiqar A Bhutta

**Affiliations:** 1 Division of Women and Child Health, Aga Khan University Karachi, Pakistan

**Keywords:** chronic disease, diabetes, hypertension, preconception, epilepsy

## Abstract

**Introduction:**

A large proportion of women around the world suffer from chronic diseases including mental health diseases. In the United States alone, over 12% of women of reproductive age suffer from a chronic medical condition, especially diabetes and hypertension. Chronic diseases significantly increase the odds for poor maternal and newborn outcomes in pregnant women.

**Methods:**

A systematic review and meta-analysis of the evidence was conducted to ascertain the possible impact of preconception care for preventing and managing chronic diseases and promoting psychological health on maternal, newborn and child health outcomes. A comprehensive strategy was used to search electronic reference libraries, and both observational and clinical controlled trials were included. Cross-referencing and a separate search strategy for each preconception risk and intervention ensured wider study capture.

**Results:**

Maternal prepregnancy diabetic care is a significant intervention that reduces the occurrence of congenital malformations by 70% (95% Confidence Interval (CI): 59-78%) and perinatal mortality by 69% (95% CI: 47-81%). Furthermore, preconception management of epilepsy and phenylketonuria are essential and can optimize maternal, fetal and neonatal outcomes if given before conception. Ideally changes in antiepileptic drug therapy should be made at least 6 months before planned conception. Interventions specifically targeting women of reproductive age suffering from a psychiatric condition show that group-counseling and interventions leading to empowerment of women have reported non-significant reduction in depression (economic skill building: Mean Difference (MD) -7.53; 95% CI: -17.24, 2.18; counseling: MD-2.92; 95% CI: -13.17, 7.33).

**Conclusion:**

While prevention and management of the chronic diseases like diabetes and hypertension, through counseling, and other dietary and pharmacological intervention, is important, delivering solutions to prevent and respond to women’s psychological health problems are urgently needed to combat this leading cause of morbidity.

## Introduction

Preconception care for women with underlying chronic diseases is very crucial. Worldwide, 60 million women of reproductive age have type-2 diabetes [1]. While diabetes has known macro- and micro-vascular complications, this increasing prevalence in women of reproductive age makes it a serious health concern for those to-be mothers and their newborns. Diabetes during pregnancy is associated with increased risk for miscarriages, stillbirth, macrosomia and obstetric complications, intrauterine developmental and growth abnormalities, birth and neonatal complications [2,3]. Strict control of blood glucose during pregnancy is necessary, however counseling, diet modification and tight glycemic control in the preconception period offer a greater benefit to maternal and newborn outcomes.

Thyroid disease is another prominent chronic illness in women of child-bearing age, second only to diabetes. Thyroid hormone imbalances during pregnancy, particularly during the first trimester, are known to cause intellectual impairment of the offspring as well as pregnancy complications including hypertension and preeclampsia, placental abruption, anemia, postpartum hemorrhage, preterm birth, low birth-weight and fetal death [4].

Phenylketonuriais an important metabolic disorder that has been associated with neurological sequelae and congenital heart defects in neonates if levels of phenylalanine are not controlled during pregnancy [5]. Therefore, the most favorable period to achieve control is before conception.

Other than the physical health of women of child bearing age, their mental health is equally important in ensuring healthy outcomes for both mother and child. Mood and anxiety disorders are highly prevalent among women of reproductive age and there is evidence that new-onset illness or a relapse is highly prevalent during pregnancy [6]. Psychiatric disorders during pregnancy have been associated with poor obstetric outcomes, higher risk of postpartum psychiatric illness, increased rates of substance abuse, and lower participation in prenatal care leading to adverse infant outcomes [7,8]. Intimate partner violence (IPV) has serious consequences for women’s psychological and physical health. Victims of IPV are at high risk of unplanned pregnancy due to sexual coercion.

Prior to taking on the challenge of supporting another life, women should be in their optimal physical and psychological health. This paper highlights the maternal and fetal risks from uncontrolled chronic diseases and potential interventions that have been effective in alleviating these risks. This paper has also assessed the risks associated with psychological health and IPV in particular and the interventions that have met with some success in dealing with these.

## Methods

This paper systematically reviewed all the literature published up to 2011 to identify studies describing the effectiveness of preconception interventions (any intervention provided to women and couples of childbearing age, regardless of pregnancy status or desire, before pregnancy or between two pregnancies, to improve health outcomes for women, newborns and children period before pregnancy and between pregnancies) and risks for preventing and managing chronic diseases and promoting psychological health for improved maternal, newborn and child health (MNCH) outcomes. Electronic databases such as PubMed, Cochrane Libraries, Embase, and WHO Regional Databases were searched to identify the experimental and observational studies on the subject. Papers were also identified by hand searching references from included studies. No language or date restrictions were applied in the search. The findings were presented at international meeting [9,10] and shared with professionals in the relevant fields of maternal and child health, following which results were updated based on current searches (through end of 2012) and expert opinion. Studies were included if they reported the effectiveness of interventions for preventing and managing chronic diseases and promoting psychological health on MNCH outcomes. The methodology is described in detailed elsewhere [11].

Two authors assessed the eligibility of studies and extracted data and judged the quality on standardized sheets. The quality of experimental studies were assessed using Cochrane criteria [12], whereas STROBE guidelines were used to assess the quality of observational studies [13]. We conducted meta-analyses for individual studies and pooled statistics was reported as the odds ratio (OR) and relative risk (RR) between the experimental and control groups with 95% confidence intervals (CI). Mantel–Haenszel pooled RR and corresponding 95% CI were reported or the Der Simonian–Laird pooled RR and corresponding 95% CI where there was an unexplained heterogeneity. All analyses were conducted using the software Review Manager 5.1 [14]. Heterogeneity was quantified by Chi^2^ and I^2^, in situations of high heterogeneity, causes were explored through sub-group analysis and random effect models were used.

## Results

**Figure 1:**
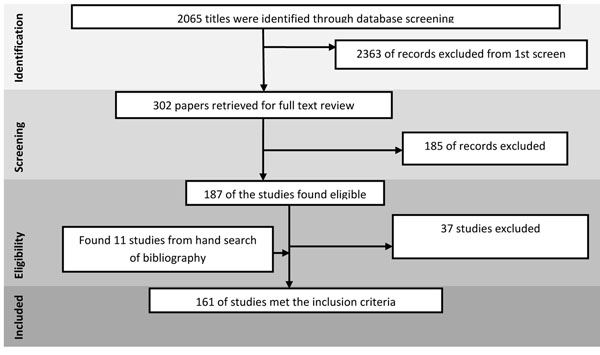
Search flow diagram

The review identified 2065 papers from search in all databases. After the initial title and abstract screening, 187 full texts were reviewed to identify papers which met the inclusion criteria and had the outcomes of our interest. One hundred and sixty one studies were finally selected for abstraction and analysis (Figure 1). Information related to each included study can be found on the following link:

https://globalmotherchildresearch.tghn.org/site_media/media/articles/Preconception_Report.pdf

## Diabetes

Diabetes continues to be an ever-increasing global problem. The prevalence of Type 2 (characterized by hyperglycemia in the context of insulin resistance and relative lack of insulin) diabetes continues to increase worldwide [15,16], especially in the low and middle income countries (LMICs) [17,18]. This in turn means more women of reproductive age in LMICs have diabetes, hence a greater number of pregnancies are complicated by the condition [19,20] putting both the mother and the fetus at an increased risk of morbidity and mortality [21]. Diabetes in pregnancy is associated with elevated rates of miscarriage [22], pre-eclampsia [23,24], preterm labor and caesarean sections [25,26] and higher rates of fetal malformation [2,3,25,27] neural tube defect, urinary tract disorder, macrosomia [28,29], birth injury [26,27,30], and perinatal mortality [31,32].

Preconception diabetic care is a multidisciplinary approach with the goal of care being to obtain the lowest possible hemoglobin A1C without significant episodes of hypoglycemia. The content for preconception care broadly includes educating the patient with regards to the disease and its interplay with pregnancy; educating the patient about self-management skills; physician-directed assessment and care of the disease and complications; counseling about diet, exercise and reproductive advice.

**Figure 2:**
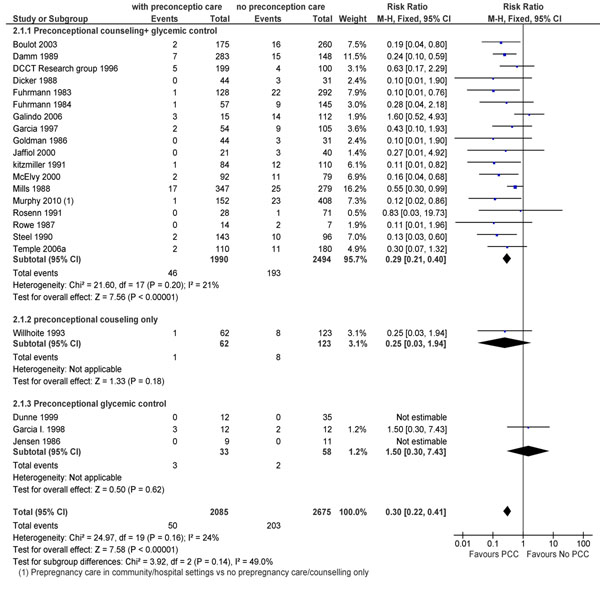
congenital malformations in preconception care versus non preconception care: evidence from observational study

**Figure 3:**
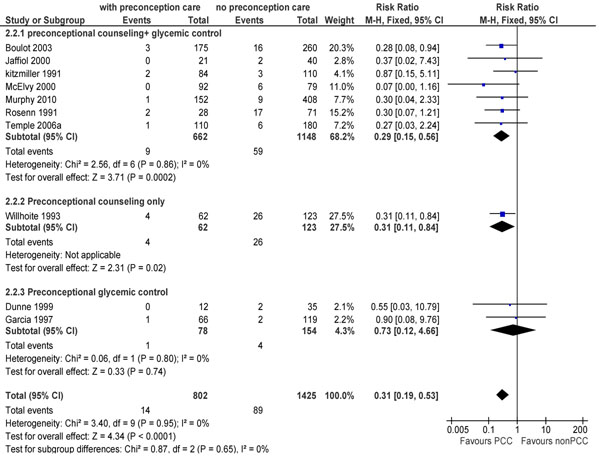
Perinatal mortality in preconception care versus non preconception care evidence from observational study

The review identified 22 observational studies [32-53] and one trial [54] that looked at various outcomes related to pre-gestational diabetes. Meta-analysis of 21 studies showed that preconception care was able to reduce the occurrence of congenital malformations (RR 0.30; 95% CI: 0.22-0.41) (Figure 2). Pooled data for the effect of preconception care on the risk of perinatal mortality was also significant (RR 0.31; 95% CI: 0.19-0.53) with counseling plus strict glycemic control leading to a 71% reduction in the events in this group compared to the standard antenatal care group (Figure 3).

When looking at pregnancy complications, the meta-analysis supported the effectiveness of preconception care in non-significantly reducing the rate of preterm delivery (RR 0.83; 95% CI: 0.62-1.12) and of caesarean sections (RR 0.97; 95% CI: 0.77-1.23) [39,41,48,50,53]. Results for other fetal/neonatal outcomes [53] and macrosomia [48,50,51] were also non-significant. The data revealed that preconception care was valuable in significantly dropping the level of HbA1C during the first trimester of pregnancy (MD -1.71; 95% CI: -2.72,-0.71) [32-34,38,40,45-47,55,56]. As hyperglycemia during the period of organogenesis leads to an increased risk of congenital malformations, this achievement of better glycemic control in the 1^st^ trimester may explain the concurrent reduction of anomalies as well as subsequent perinatal death. A single study by Heller et al. [54] showed a weak non-significant effect of preconception insulin in reducing the 1^st^ trimester HbA1C as compared to commencement of insulin in early pregnancy (MD -0.10; 95% CI: -0.27, 0.06).

## Epilepsy management

Epilepsy is a condition in which a disruption of the normal electrochemical activity of the brain results in seizures. Women with epilepsy during their child-bearing years not only face the possible risk for adverse pregnancy outcome as a result of the teratogenic effects of antiepileptic drugs upon [57,58] but also the potential effect of maternal seizures on the developing fetus [59-61]. Most women with epilepsy have no change in seizure frequency during pregnancy but about 15-33% report increased episodes of seizures during pregnancy [62]. This may be due to a change in the pharmacokinetics of the anti-epileptic drugs [63] or due to the hormonal changes occurring in pregnancy [64]. Unplanned pregnancies rates in women are high but these may be even higher in women with epilepsy because antiepileptic drugs interfere with hormonal contraception [65]. Different drugs lead to different types and different rates of anomalies, with the highest rates being associated with valproate [66,67].

Preconception care of women with epilepsy includes a careful revision of each case to ascertain the diagnosis, the need for continued anti-epileptic drug therapy, selection of suitable drugs with optimization of the dosage, and prescription of folic acid to prevent neural tube defects.

We found one study [68] that assessed the effectiveness of preconception counseling in women with epilepsy reported that none of the 85 women who were counseled before pregnancy had an abnormal fetus in the subsequent pregnancy as compared to almost 19% of the women who did not receive any preconception counseling (as they were already pregnant) who had an abnormal fetus (with 3 pregnancy terminations). One patient in the counseled group had an early miscarriage, followed by a normal subsequent pregnancy, and 1 had a preterm birth compared to 3 preterm births in the control group. They also showed that post-counseling 71% of women with epilepsy used a single drug and none used >2 drugs as compared to 32% and 20% respectively in the control group. Most of the counseled women used carbamezapine/lamotrigine compared to the control women with epilepsy, 41% of whom used valproate.

## Management of phenylketonuria

Phenylketonuria (PKU) is caused by the deficiency of phenylalanine hydroxylase which is required to essential amino acids, phenylalanine (phe), to tyrosine [69]. These women are advised to consume phe free food to live a normal life. These women during pregnancy require appropriate management as poor disease control is associated with a multitude of fetal consequences like facial dysmorphism, microcephaly, developmental delay, learning difficulties and congenital heart disease [69,70].

This review accumulated evidence from current literature on the effect of maternal PKU on the pregnancy outcome, specifically of preconception levels of phenylalanine. The review also looked for any preconception intervention which worked in lowering the maternal, newborn and child health (MNCH) risks associated with poorly controlled phenyalanine levels. Preconception care of women with PKU consists of counseling regarding the fetal risks (facial malformations, growth deficits, micorcephaly) associated with the disease, commencement of a phenylalanine restricted diet, attaining safe phenylalanine levels (100 [71] –360μmol/L [72] or <6mg/dL [73,74] atleast 3 months before conception; and maintaining them throughout gestation). When counseling patients great importance has to be put on the need for effective contraception till such safe levels are reached.

The review identified seven studies [70,75-80]. Rouse et al. [70] in a cohort of women with blood Phenylalanine levels >240umol/L found that mean phenylalanine levels at 4 to 8 weeks gestation predicted congenital heart defect (P<0.0001). They also found that each abnormality increased in frequency as Phenylalanine control was delayed. The percentage of offspring with >3 dysmorphic features (49% overall) was related to time of inadequate maternal Phenylalanine control (P=0.002), increasing from 19% in offspring of mothers in control before pregnancy to 62% when control was not achieved before 20 weeks’ gestational age. The frequency of offspring with microcephaly was significantly related to time of maternal Phenlalanine control (P=0.001)- in women who were preconceptionally treated with good control, microcephaly occurred in only 3.6% of the pregnancies [5].

From current literature the effect of a preconception dietary intervention was analyzed for growth of the fetus. The analysis showed that a strict preconception diet was significantly associated with an increment in mean birth weight (MD 0.60; 95% CI: 0.39-0.82) and increase in head circumference (MD 3.20; 95% CI: 2.37-4.03) compared to no dietary restrictions [80]. Improved infant growth markers were also associated with following a strict preconception diet in other studies [75,79]. Koch et al. [75] reported that a preconception diet led to a 1^st^ trimester PHe level of 500umol/L compared to 641umol/L in those on a post conception diet. Maillott et al. [76] also reported a significant decrease in 1^st^ trimester mean PHe level in those on a preconception diet versus a post-conception diet [248.8+/-86.6 compared with 493+/-289.4mol/L; P<0.0001].

## Addressing thyroid disorders preconceptionaly

Women of child bearing age may suffer from hypo- or hyper-function of the thyroid gland, more often than not due to an autoimmune process. Hypothyroidism during pregnancy is known to lead to adverse maternal (gestational hypertension and pre-eclampsia [81], postpartum hemorrhage, abortion [4] and preterm delivery [82,83]), fetal (congenital anomalies, growth retardation [84], perinatal death) [82] and neonatal consequences (cognitive disorders) [85]. Literature on the association between thyroid disease during pregnancy and preterm delivery is most abundant, with most attributed to autoimmune thyroid disease [86-89]. Among the most frequent complications are the hypertensive disorders of pregnancy, also reported are spontaneous abortion and preterm delivery [90].

While literature on the effect of thyroid status on maternal, fetal and neonatal effects is abundant, much work still needs to be done with regards to the effect of preconceptional thyroid status on these outcomes. Many recommend attainment of a TSH <2.5mU/L before the start of pregnancy. Since purely preconception literature was unavailable, the review looked at the effect of peri-conceptional interventions addressing adverse pregnancy related outcomes and even those studying the effect of the disease and treatment on MNCH outcomes. Content of preconception care for women with thyroid disorders consists of a thorough assessment of the disease status, advice on the achievement of a euthyroid status well before conception, counseling about the pregnancy-related risks associated with thyroid dysfunction. Medications need to be adjusted in order to have optimal thyroid function and the importance of useful contraception should be stressed upon till such a time.

**Figure 4:**
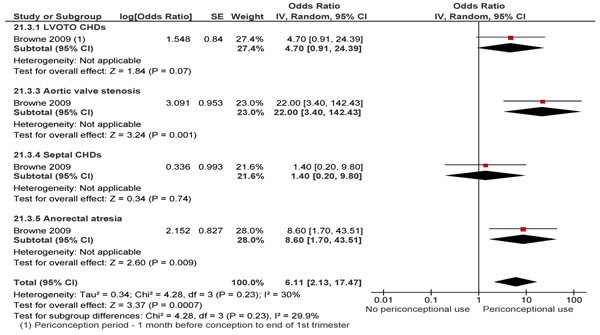
Periconception use of anti-thyroid medications and Birth defects evidence from observational study

Thyroid status at the time of conception plays an important role. According to the study by Abalovich et al. [85] none of the women who were euthyroid at the time of conception experienced preterm deliveries. Mestman et al. [91,92] underscores the importance of pre-pregnancy counseling for hyperthyroid women and the use of contraception until achievement of a euthyroid status before conceiving. Earl et al. [93] found no interventions for the prevention and treatment of hyperthyroidism during pregnancy. Their result for usage of antithyroid drugs (ATD) was inconclusive due to the small potential risk of adverse fetal effects of methimazole and maternal effects of propylthiouracil. Another study reports that both ATDs are equally effective and safe in the treatment of hyperthyroidism in pregnancy [94]. Periconception use of ATD was however; shown to significantly increase the rates of selected birth defects (Figure 4) [95].

Browne et al. [95] also reported an association of periconception thyroxine and selected birth defects. Rotondi et al. [96] conducted a trial on the preconception adjustment of levothyroxine and found that it may lead to adequate thyroid function in the 1^st^ trimester; however they did not look at any MNCH outcome. Results suggest that in hypothyroid women anticipating pregnancy (with serum TSH in the lower quartile of normal range), the pre-conception adjustment of L-T4 doses may result in adequate maternal thyroid function up to the first post-conception evaluation [96].

Vaquero et al. [97] reported that in thyroid supplementation group (66mg of thyroid extract, started before conception and continued until the 20th week) among patients with thyroid antibodies, 13 out of 16 pregnancies (81.2%) ended in live birth. Only one pregnancy loss occurred among patients with a mild underlying thyroid pathology treated with thyroid replacement therapy.

## Systemic lupus erythromatoses and other connective tissue diseases

Systemic lupus erythematous (SLE) predominantly affects women in the childbearing age group, and thus the effect of pregnancy on the disease and vice versa is an important consideration in the management of these patients. Despite all the advances in understanding the disease pathology and management options pregnancy in lupus is still considered to be a high-risk pregnancy [98]. There is a higher rate of fetal loss, pre-term delivery and IUGR in lupus pregnancies [98-100]. Pre-existing hypertension or renal dysfunction further increases the risk of pre-eclampsia and pregnancy-induced hypertension (PIH) [100-102]. Several studies have found the frequency of fetal loss to vary between 11-24% [99,103-106]. While some studies advocate that active disease increases the risk of fetal loss [104,107,108], other studies show no statistically significant difference between pregnancies in women with active lupus and those in women with inactive lupus [109,110]. Active disease at conception is a known predictor of poor outcome [102,109,111]. A flare during the year prior to conception pointed to increased risks of a flare again during pregnancy [112,113].

SLE is a prime example of an autoimmune disorder. The review used this disease to study the possible effects of autoimmunity on MNCH outcomes. The review also looked at the effects of treatment modalities for SLE and how, if any available intervention (like counseling, behavioral programs) targeting such women improved the pregnancy outcomes.

**Figure 5:**
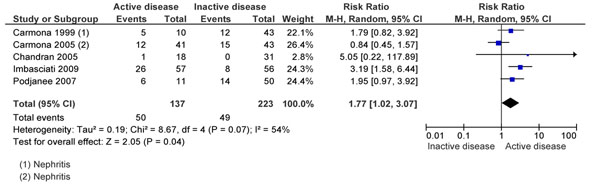
Preconception disease activity and disease flares during pregnancy evidence from observational study

The review found a number of observational studies looking at the effect of active disease in the preconception period on pregnancy related outcomes [77,102,108,109,114-128]. The analysis showed that preconception active SLE was associated with multiple maternal and fetal/neonatal outcomes. An active disease increased the risks of gestational flares by 77% (P 0.04) (Figure 5) [117,123,125-127]. There was an over three-fold increase in the risk of developing PIH if the disease was active (specifically with nephritis) before pregnancy (p=0.002); no association was found with risk of preeclampsia. There was also a significant rise in the preterm deliveries if the disease was not in remission before conception (RR 1.71; 95% CI: 1.18-2.48); this risk was further increased by 13% if the woman suffered from active nephritis pre-pregnancy.

**Figure 6:**
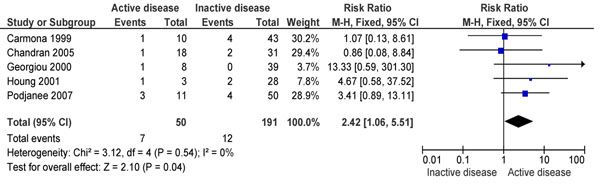
Preconception disease activity and perinatal mortality evidence from observational study

Coming to adverse SLE related fetal/neonatal outcomes, it was seen that a positive disease activity in the preconception period significantly increased perinatal mortality (RR 2.42; 95% CI: 1.06, 5.51) (Figure 6) [102,108,117,123,125,127,128]. No association was seen with either spontaneous abortions (RR 3.26; 95% CI: 0.58-18.14) [108,123] or restricted fetal growth (RR 0.61; 95% CI: 0.16-2.28) [127]. Similar findings were reported by Smyth et al. [129].

## Other chronic conditions

**Chronic hypertension and heart disease** – pregnancies complicated by chronic hypertension are associated with increased risk of hypertensive disorders of pregnancy and other organ dysfunctions as well as increased fetal risks of preterm birth, intrauterine growth retardation, fetal loss, hypospadias and abruption placenta [130].

Romunstad et al. [131,132] found a significant association between prepregnancy systolic as well as diastolic blood pressure and low birth weight (LBW). Magnussen et al. [133] found systolic blood pressures of greater than 130mmHg to increase the risk of pre-eclampsia by more than 7 times. Because there is an increasing burden of unplanned pregnancies, fetal exposure to antihypertensive medications might occur before a woman knows she is pregnant. Caton et al. [130] studied the effect of periconception use of anti-hypertensives and found a positive association with the occurrence of hypospadias (OR 1.90; 95% CI: 1.00-3.61) [130], with a non-significant increase with exposures only to antiadrenergic agents at any time between 1 month preconception and the fourth month of pregnancy. Hameed et al. [134] found an increased risk of spontaneous abortion, cardiac anomaly (4-14%) in the presence of maternal congenital heart disease (CHD).

**Asthma** - Research demonstrates that women with severe asthma prior to pregnancy is more likely to worsen during pregnancy. This reinforces the importance of adequate asthma control prior to conception [135]. Asthma that is not adequately controlled during pregnancy can result in serious maternal complications (preeclampsia, hypertension, and hyperemesis gravidarum) [136] as well as increased fetal complications (stillbirth and infant death, neonatal hypoxia, intrauterine growth retardation, premature birth, and LBW) [137]. It is observed that the dangers of uncontrolled asthma are greater than the risks of indispensable asthma medications. Whereas oral corticosteroid use in the first trimester has been associated with reduced birth weight, an increased risk of preeclampsia, and an increased risk of oral clefts [138,139]. Analysis showed that periconception use of asthma medications was significantly linked to a greater risk of gastroschisis (OR 2.12; 95% CI: 1.39-3.24) [140] especially the use of bronchodilators which significantly doubles the risk.

**Chronic renal disease**- Adverse pregnancy outcomes associated with maternal renal disease include preeclampsia, anaemia, chronic hypertension, caesarean delivery, preterm delivery, fetal growth restriction, and increased fetal loss and stillbirth [141,142]. Renal hypertension is associated with a 10-fold increase in fetal loss compared to women with spontaneously or therapeutically normal blood pressures [143].

**Headache** – frequent pre-pregnancy headaches were found to be statistically significantly associated with poor mental health in the first 3 months of gestation as well as with antepartum depression [144].

**Multiple sclerosis** - Vukusic et al. 2004 [145] reported that women with greater disease activity in the year before pregnancy have a higher risk of relapse in the postpartum 3 months (OR 1.3, p 0.04).

## Medication use

Medication usage among pregnant women and women of reproductive age is common. It has been estimated that more than 80% of pregnant women take over-the-counter or prescription drugs during pregnancy [146]. National surveys among women of reproductive age document that chronic conditions often requires the ongoing administration of medications for maintenance are not uncommon among women of reproductive age [147]. As maternal age and body mass index increases, it is likely that an even greater proportion of women who are planning a pregnancy or who could become pregnant will have chronic diseases that necessitate prescription medications. Generally, medication carries a risk of unwanted side-effects which may have profound impacts during pregnancy.

The aim was to look for studies assessing interventions dealing with the repercussions of various medications being frequently used by women. Regular medications being used by women suffering from chronic diseases are covered in the sections of their respective disorders. Studies particularly addressing the deleterious effects of medications, on the health of both the mother and the fetus, when taken in the period before conception were assessed. The review found studies assessing the effect of use of weight-loss drugs, oral contraceptives and vasoactive agents.

**Weight loss drugs -** Analysis of the effect of periconception use of weight-loss drugs showed a significant association with overall higher rates of congenital anomalies (OR 1.59; 95% CI: 1.33-1.89) [148]. This association was stronger for congenital heart defects with an 88% increase in incidence of Dextro-Transposition of the great arteries and a 58% increase in the incidence of Left Ventricular Outflow Tract Obstruction (LVOTO) (OR 1.88; 95% CI: 1.33-2.65); (OR 1.58; 95% CI: 1.22-2.04) respectively. Bitsko et al. [148] reported the association with ‘Aortic Stenosis’ to be highest among the LVOTO defects (OR 1.2; 95% CI: 0.5-3.1).

**Figure 7:**
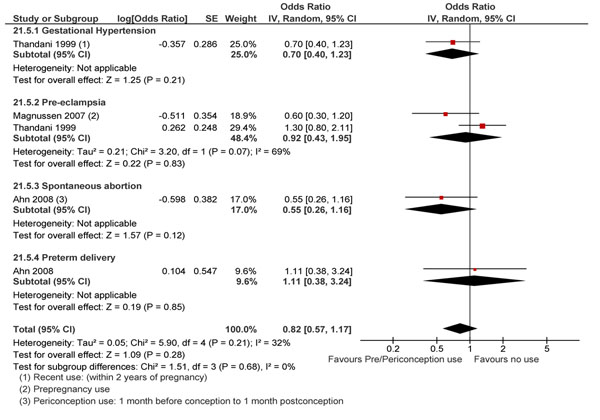
Pregnancy outcomes of Pre/Periconception use of oral contraceptives evidence from observational study

**Oral contraceptive pills (OCPs) –** several studies identified that reported OCPs and maternal and fetal outcomes [133,149-151] No significant association was found between pre/peri-conception use of oral contraceptives and gestational hypertension [150], pre-eclampsia [133,150], preterm delivery, spontaneous abortion (Figure 7), however periconception use of oral contraceptive pills (OCP) lead to an almost three-fold increase in the risk of Down’s in infants (RR 2.71; 95% CI: 1.48-4.95) [151].

**Vasoactive substances -** Werler et al. [152] reported aspirin use in the periconception period led to a significantly greater risk of amniotic bands (OR 2.5; 95% CI: 1.4-4.6). Vasoconstrictor and decongestant use led to a higher incidence of transverse limb defects (OR 1.4; 95% CI: 1.1-2.0) and (OR 1.7; 95% CI: 1.2-2.3) respectively.

**Anti-hypertensive medications** - Caton et al. 2008 [130] observed slight to moderate elevations in the risk of severe hypospadias for maternal untreated hypertension (OR 2.1; 95% CI: 1.6-2.9) and antihypertensive medication use during 1 month preconception through pregnancy month 4 (OR 1.4; 95% CI: 0.7-2.9).

**Bronchodilators** – Lin et al. 2008 [140] reported significant association of maternal bronchodilator use with gastroschisis (OR 2.06, 95% CI: 1.19, 3.59).

**Thyroxin –** Few studies [95,96,153] reported non-significant association with birth defects (OR 1.7; 95% CI: 1.0-2.7).

**Any illness/common cold -** A study by Krapels et al. 2006 [154] displayed an association of any maternal illness and common cold in periconception period (3 months before conception to 3 months after) with orofacial defects. Cleft lip with or without cleft palate increases by 1.7 times (95% CI: 1.2-2.5) and cleft palate only by 1.5 times (95% CI: 0.8-2.6).

## Mental health

With the current prevalence of psychiatric illnesses, there is a significant risk of women’s antenatal and postpartum periods being made difficult with the onset or recurrence of a psychiatric illness. Evidence suggests that depression and anxiety during pregnancy and postpartum severely impact family life, the mother-infant relationship, and the future mental health of the child [6-8,155]. A large meta-analysis stated that up to 18% of women experience depressed mood during pregnancy [156]. A Brazilian study noted that common mental disorders, in general, were autonomously related with LBW and post-traumatic disorder (PTD) in pregnant teenagers javascript:newshowcontent('active','references');[157]. Maternal antenatal depression generally has been highly correlated with PTD [158-160]. Similarly, depression also appears to be a significant risk factor for LBW [159,161,162]. Depression also has noteworthy associations with miscarriage, antepartum hemorrhage, greater uterine artery resistance and a higher risk of operative deliveries [161]. Additional risks are associated with the medications being used to treat depression. Selective serotonin reuptake inhibitor (SSRIs) has been linked with earlier gestational age and lower birth rate [163,164]. Other studies also suggest first trimester exposure to SSRIs increasing the risks restricted fetal growth [165,166].

Bipolar disorder is a severe recurrent illness that is associated with high rates of morbidity and mortality in the absence of adequate treatment. Manic episodes may be associated with increased risky behaviors such as sexual activity or substance use, which could affect health during pregnancy as well as lead to a significant risk of unintended pregnancies [167]. Patients with bipolar disorder have a very high risk of comorbid alcohol or substance abuse disorders – reaching up to 60% in some studies - which could have direct adverse impacts on fetal outcomes [168,169].

The review assessed the effect pre-existent psychiatric conditions in women in the preconception period on maternal and fetal morbidity and mortality. Included were more prevalent conditions like mood disorders as well as conditions like schizophrenia. The review looked primarily at the risks and benefits, to the mother as well as her unborn child, of continuing or changing or even discontinuing the psychotropic regimens for the above mentioned disorders.

While the effect of psychiatric conditions and their relative treatment during pregnancy has been widely studied [170-189], there is a serious lack of evidence of how pre-pregnancy disease and psychotropic drugs may affect pregnancy. The review found that pre-pregnancy depression is significantly related to preterm births (OR 1.04; 95% CI: 1.02-1.07) [171] and adolescent depression per say was significantly associated with an increased risk of miscarriages (OR 2.25; 95% CI: 1.12-4.50) [172]. When assessing for maternal morbidity, adolescent depression was positively associated with suffering from intimate partner violence (IPV) (OR 3.47; 95% CI: 1.11-10.84) but not with sexually transmitted infections (STIs) (OR 1.50; 95% CI: 0.83-2.72) [172]. Silverman et al. [173] concluded that a pre-existing psychiatric condition was one of the best predictors of development of post-partum depression. Literature also showed that a pre-pregnancy psychotic or bipolar illness substantially increased the risk of a postpartum psychotic or bipolar event [174]. The search for the effect of maternal bereavement on neonatal/infant health revealed that loss of a close relative in the 7-12 months before conception did not increase the risks of autism, epilepsy or febrile seizures in the infant. However, loss of a child or spouse in the 6 months preceding conception was positively associated with attention deficit hyperactivity disorder (ADHD) in the male child, childhood obesity and congenital malformation.

**Figure 8:**
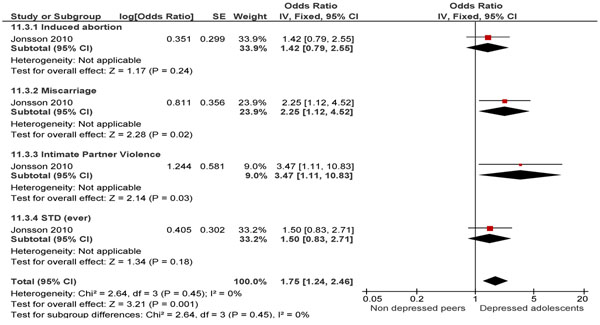
Morbidities associated with depression in adolescents: evidence from observational study

Interventions specifically targeting women of reproductive age suffering from a psychiatric condition show that group-counseling [175] and interventions leading to empowerment of women have reported lowering of depression in these women but the results so far have not been significant (economic skill building: MD -7.53; 95% CI: -17.24, 2.18; counseling: MD-2.92; 95% CI: -13.17, 7.33) [176]. Interventions teaching coping skills or based on stepwise facilitation seem to significantly lower depression levels and these lowered levels were persistent at the 1-yr follow-up [177]. However, morbidities associated with depressions are higher (Figure 8).

Women with serious mental illnesses are at a greater risk of having had >1 sexual partner or having been raped and are hence more likely to have unplanned, unwanted pregnancies [190]. Their support system has been reported to be generally lacking [191]. They have a greater possibility of engaging in risky behavior during pregnancy (substance abuse, suicide attempts) or of being abused [192]. All this makes it imperative for their physicians to not only screen vigorously for such cases but also to provide comprehensive family planning and contraceptive counseling as well as attach them to relevant support systems.

## Intimate partner violence

Irrespective of demographics, women around the globe have been subjected to IPV. IPV against women is a major public health concern as it adversely affects both the physical, mental and reproductive health of a woman and that of the newborn. physical abuse by a partner at some point in life was reported by 13–61% of women of 49 years of age and sexual violence by a partner was reported by 6–59% of them [190]. Violence during pregnancy has been associated with poor health outcomes including increased risk of preterm labor [191], antepartum hemorrhage [192], LBW infants [191], fetal loss [193-195], STIs [196] and post-partum depression [197]. Coker et al. [198] reported that women who ‘ever experienced’ IPV were more than twice as likely to suffer from various kinds of physical and mental health problems. Having experienced IPV is associated with a higher occurrence of unwanted pregnancies [199-201], gynecologic morbidity [202-204] and involvement in risky sexual behaviors [202,204,205]. Data suggests that intensive advocacy interventions may improve the quality of life where as brief advocacy interventions improve safe behaviors [206].

The content of preconception care for women suffering from IPV includes firstly identifying such women, which can be effectively done by asking all women about their experiences of violence from any source, at any point in life. Their condition needs to be evaluated and their injuries treated. Women suffering from IPV need to be informed about the significant harm to the mother, the fetus, and the newborn infant that such abuse can potentially cause and hence of the crucial role of contraceptives. They need be counseled for the psychological trauma that they’ve suffered from. Finally they need to be referred to an agency/support group that specializes in dealing with such cases. Sexual violence specifically in adolescents is dealt with in the section on ***‘Adolescents’*** (Reference to paper on adolescent health).

**Figure 9:**
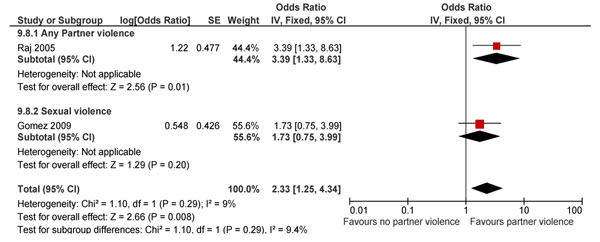
IPV and risk of unintended pregnancies (in women who have undergone IPV in the last 1 year: evidence from observational study

Majority of the reviewed studies on effect of IPV exposure were in women in the general population and were risk aversion studies [191-197,200-205,207-242], while few were intervention studies [176,177,243-253]. From the analysis it was found that IPV positively led to unintended pregnancies (OR 2.33; 95% CI: 1.25-4.34) (Figure 9) [215,218].

No association was found between IPV and condom use in women [196,202,218]. A significant increase in gynecologic morbidities was reported in women suffering from IPV (OR 1.45; 95% CI: 1.13-1.85) [196,203] and rates of STIs were non-significantly raised by around 2 folds in these women (RR 1.89; 95% CI: 0.65-5.47) [202,233]. Gynecologic morbidity increased significantly with any spousal abuse (OR 1.89; 95% CI: 1.23-2.91); combined physical plus sexual violence led to a 72% increase (P 0.04).

**Figure 10:**
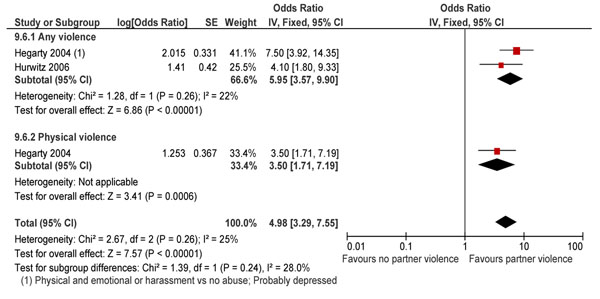
IPV and risk depression (in women who have undergone IPV in the last 1 year: evidence from observational study

With regards to a woman’s physical and mental health, IPV had serious detrimental effects in those abused. Ruiz et al. [254] reported that women who had experienced physical, psychological and sexual violence were twice as likely to suffer a chronic disease as those who have not experienced abuse (OR 2.03; 95% CI: 1.18-3.51), especially diseases other than hypertension, diabetes and asthma (OR 2.57; 95% CI 1.38-4.77) [229], and fetal loss [193]. IPV leads to a towering five-fold increase in depression among the victims (P<0.00001) (Figure 10) [222,224] and a two-fold increase in impairment of mental health in the past month only (RR 2.08; 95% CI: 1.70-2.55) [232,233]. Abuse also makes these women 7 times more likely to contemplate suicide [222].

Interventions targeting IPV have mainly looked at behavioral therapies. These studies have yielded non-significant effects on the occurrence of new events of violence post-treatment. A meta-analysis of 4 trials comparing cognitive behavior therapy (CBT) versus no intervention showed a reduction favoring the intervention group [234]. Behavioral couple’s therapy, when compared to gender specific treatment, showed greater reductions in post-treatment aggression and recidivism rates, especially multiple couple’s group sessions. A dual intervention targeting both IPV and substance abuse showed decreased rates of both in the intervention group (RR 0.71; 95% CI: 0.37-1.38) [188].

Interventions focusing on empowerment of women have been employed to reduce these risks, but their role in decreasing the rate of IPV have so far not been significant. A pilot on the effectiveness of an intervention to reduce male partner reproductive coercion was associated with a large reduction in pregnancy coercion among women who had recently experienced IPV (OR: 0.29; 95% CI: 0.09-0.91) [255].

## Discussion

Preconception care (diet and exercise counseling, and a stringent glycemic control) for women with preexisting diabetes is effective in addressing the ever-increasing rates of adverse fetal consequences (congenital malformation, perinatal mortality) as well as serious maternal outcomes (preterm labor, level of maternal HbA1c in the first trimester of pregnancy). This review identified significant impact of preconception diabetic care on reducing congenital malformation and perinatal mortality. This finding is in line with the results of some previous reviews [25,256] with the differences being attributed to inclusion of studies with a low to moderate level of bias. The problem however lies in the fact that a substantial number of women with diabetes do not access such preconception care interventions and continue to have unplanned pregnancies with deleterious MNCH results. Since less than 30% of those with diabetes present for preconception care, every office visit of every female diabetic adolescent or woman of childbearing age should be regarded as a preconception care visit. Also with more women having children in their later years, screening for type 2 diabetes among women of childbearing age becomes more important. Future research, however, needs to aim at evaluating the effectiveness of preconception care on the incidence of other MNCH outcomes like caesarean sections, spontaneous abortions, via proper trials. What it needs more is to find ways of successfully integrating preconception care into the routine care of all women of reproductive age suffering from diabetes.

On the other hand, preconception management is the cornerstone for epilepsy care in women with epilepsy. What is recommended is a multidisciplinary approach, involving the patient’s primary care physician, an obstetrician who specializes in high-risk pregnancies, and a neurologist. Women with epilepsy should be reviewed before planning a pregnancy in order to optimize therapy before conception. Ideally changes in antiepileptic drug therapy should be made at least 6 months before planned conception, if possible. All women with epilepsy should be persuaded to begin folic acid supplementation during reproductive years and continue throughout pregnancy. A recent survey [257] reported that women with epilepsy are not getting the advice they need on issues relating to contraception and pregnancy. This point was also conformed in another study [258] which showed that physicians managing women with epilepsy did not place adequate emphasis on preconception care. The current evidence for preconception counseling is encouraging but not conclusive and requires further thorough investigation. Effective elements of counseling or mode of delivery need to be identified via future research. Trials should be conducted to evaluate the value of counseling or other behavioral interventions in the preconception period in reducing clinically relevant outcomes. While there is an extensive support for the pre-conception counseling of all women of child-bearing years suffering from epilepsy, there is a dearth of evidence evaluating the efficiency of such an intervention in dealing with adverse pregnancy outcomes of the disease and its treatment.

Given the complications of the maternal PKU syndrome, a systematic approach to those intending to get pregnant is required. The analysis revealed a significant positive effect of strict dietary control in the preconception period and improved growth parameters in the newborn. Studies have also reported a decrement in other fetal risks associated with the disease after attainment of an adequate control of phenylalanine levels in the 1^st^ trimester, brought about by following a stringent dietary plan before pregnancy. There is evidence that a preconception phenylalanine-restricted diet works, however what is needed now is to finalize a preconception protocol for women with PKU and implement it on a larger scale for better coverage. An absolute dearth of evidence was also identified in women with thyroid dysfunction. However, logic dictates that ensuring maternal biochemical euthyroidism in the first trimester, when the fetus is most responsive tomaternal thyroxine, might optimize fetal outcome. To achieve this target those already suffering from thyroid dysfunction need to be re-evaluated before they plan to conceive, their treatment regimens need to be re-adjusted and they need to be counseled about the probable risks to both lives that an unachieved euthyroid status may lead to. Future research not only needs to find the missing link between thyroid function before conception and a fall in associated MNCH morbidities, it also needs to focus on how to achieve this in women with thyroid disorders who want to conceive.

Pregnancy is safe in most lupus patients who conceive while the disease is inactive; however pregnancy statistically increases SLE activity. Active SLE prior to pregnancy is associated with a less favourable maternal and fetal outcome and conception should hence be avoided, if possible. The analysis showed that an active disease status in the preconception period significantly increased the risks of gestational flares by 77%, PIH by over three folds, preterm deliveries by twice as much and perinatal mortality by over two-folds. No association was found with preeclampsia, fetal growth restriction or spontaneous abortions. These findings highlight the importance of a preconception intervention to address the reproductive issues in women suffering from SLE. Similarly, several drugs used by women with lupus have been contraindicated during pregnancy for their adverse effects on maternal and fetal outcomes.

This review of the literature found important evidence pertaining to the periconception use of certain drugs used regularly for chronic disorders or other purposes. Anti-asthmatics, especially bronchodilator use, in the periconception period led to a more than two-fold increase in the incidence of gastroschisis. Weight-loss drugs led to a 58% increase in the risk of congenital malformations, especially congenital heart defects. OCPs led to a non-significant increase in various pregnancy and fetal outcomes. Vasoactive substances, like aspirin, decongestants and vasoconstrictors were associated with limb defects. It is therefore, advisable to women to take these drugs only when prescribed by doctors after thorough assessment of history and examination of potential side effects.

Mental health conditions are prevalent among women of reproductive age and a substantial proportion goes untreated. Due attention is being paid to screening for and treating psychosocial issues during pregnancy and post-partum but non-pregnant women are being neglected in this regard. There is a deficiency of evidence associating the status of disease and treatment in the preconception period with adverse MNCH effects. This explains the lack of literature on effective interventions targeting such women, implementation of which would be a task of its own. Interventions already proved efficacious in pregnancy should also be evaluated for women before pregnancy. IPV, on the other hand, is a serious, widely prevalent issue. Apart from being violations of human rights, acts of violence profoundly damage the physical, sexual, reproductive, emotional, mental and social well-being of not only individuals but families. IPV has adverse effects on women, leading to an increase in unplanned pregnancies, gynecologic infections and probable fetal loss Current interventions for reducing IPV and related morbidities have mainly looked at behavioral therapies. Behavioral couple’s therapy has shown greater reductions in post-treatment aggression and recidivism rates, especially multiple couple’s group sessions. Although these interventions may not have shown significant effects yet, there is every reason to believe that thorough outcome evaluations of present programmes along with development of new programs based on sound supposition and identified risk factors will translate into a swift expansion in the near future.

Although the review identified the impact of majority of chronic and mental diseases on MNCH outcomes, it was, however, unable to gather evidence on preconception respiratory diseases such as cystic fibrosis, endocrine problems such as ovarian syndrome and women with cancers. This was mainly because of dearth of literature on these topics particularly from preconception period.

## Conclusion

Provision of care to high risk women for chronic medical conditions is as important as any other general health promotion. Delaying and achieving optimal timing of a pregnancy is often an important component of the preconception care of women with medical conditions. Since majority of the pregnancies are unplanned, therefore, reproductive planning and contraceptive considerations for women of reproductive age with chronic medical conditions should be discussed early after diagnosis. IPV is a serious, widely prevalent issue. Apart from being violations of human rights, acts of violence profoundly damage the physical, sexual, reproductive, emotional, mental and social well-being of not only individuals but families. IPV has untoward effects on women, leading to an increase in unplanned pregnancies, gynecologic infections and probable fetal loss. Abuse also leads to grave impairment of the physical and mental health of the victims.

## Competing interests

We do not have any financial or non-financial competing interests for this review.

## Peer review

Peer review reports are included in additional file 1.

## Supplementary Material

Additional file 1 Peer review reports. Click here for file

## Declarations

This article has been published as part of *Reproductive Health* Volume 11 Supplement 2, 2014: Preconception interventions. The full contents of the supplement are available online at http://www.reproductive-health-journal.com/supplements/11/S3. Publication charges for this collection were funded by the Partnership for Maternal, Newborn & Child Health (PMNCH).

## References

[B1] Hunt KJ Schuller KL The increasing prevalence of diabetes in pregnancy Obstet Gynecol Clin North Am 2007 34 2 173 199 17572266 10.1016/j.ogc.2007.03.00 PMC2043158

[B2] Kitzmiller JL Buchanan TA Kjos S Combs CA Ratner RE Pre-conception care of diabetes, congenital malformations, and spontaneous abortions Diabetes Care 1996 19 5 514 541 8732721 10.2337/diacare.19.5.514

[B3] Ray JG Vermeulen MJ Meier C Wyatt PR Risk of congenital anomalies detected during antenatal serum screening in women with pregestational diabetes Qjm 2004 97 10 651 15367735 10.1093/qjmed/hch107

[B4] Casey BM Dashe JS Wells CE McIntire DD Byrd W Leveno KJ Cunningham FG Subclinical hypothyroidism and pregnancy outcomes Obstetrics & Gynecology 2005 105 2 239 15684146 10.1097/01.AOG.0000152345.99421.22

[B5] Koch R Friedman E Azen C Hanley W Levy H Matalon R Rouse B Trefz F Waisbren S Michals-Matalon K The international collaborative study of maternal phenylketonuria: status report 1998 European journal of pediatrics 2000 159 14 156 160 11043164 10.1007/pl00014383

[B6] Cohen JA Treating traumatized children: Current status and future directions Journal of trauma & dissociation 2005 6 2 109 121 16150673 10.1300/J229v06n02_10

[B7] Burt SA McGue M Krueger RF Iacono WG How are parent–child conflict and childhood externalizing symptoms related over time? Results from a genetically informative cross-lagged study Development and Psychopathology 2005 17 01 145 165 15971764 10.1017/S095457940505008X PMC2245887

[B8] Halbreich U The association between pregnancy processes, preterm delivery, low birth weight, and postpartum depressions--the need for interdisciplinary integration American journal of obstetrics and gynecology 2005 193 4 1312 1322 16202720 10.1016/j.ajog.2005.02.103

[B9] Dean S Rudan I Althabe F Girard AW Howson C Langer A Lawn J Reeve M-E Teela KC Toledano M Setting research priorities for preconception care in low-and middle-income countries: aiming to reduce maternal and child mortality and morbidity PLoS Med 2013 10 9 e1001508 24019762 10.1371/journal.pmed.1001508 PMC3760783

[B10] WHO WHO Meeting to develop a global consensus on preconception care to reduce maternal and childhood mortality and morbidity 2012 Geneva: World Health Organization Headquarters

[B11] Dean SV Lassi ZS Imam AM Bhutta ZA Preconception Care: closing the gap in the continuum of care to accelerate improvements in maternal, newborn and child health Reproductive Health 2014 10.1186/1742-4755-11-S3-S1 PMC4196556 25414942

[B12] Higgins JPT, Green S Cochrane Handbook for Systematic Reviews of Interventions. Version 5.0.0 [updated February 2008] The Cochrane Collaboration 2008 Available from http://www.cochranehandbook.org

[B13] Brand RA Editorial: standards of reporting: the CONSORT, QUORAM, and STROBE guidelines Clin Orthop Relat Res 2009 467 6 1393 1394 19296187 10.1007/s11999-009-0786-x PMC2674183

[B14] Review Manager (RevMan). [computer program]. Version 5.0: 2008 Copenhagen: Nordic Cochrane Collaboration

[B15] Passa P Diabetes trends in Europe Diabetes/Metabolism Research and Reviews 2002 18 S3 S3 S8 12324978 10.1002/dmrr.276

[B16] Fleming DM Cross KW Barley MA Recent changes in the prevalence of diseases presenting for health care The British Journal of General Practice 2005 55 517 589 16105366 PMC1463227

[B17] World Health O Diabetes Action Now: An Initiative of the World Health Organization and the International Diabetes Federation 2004 World Health Organization

[B18] Al-Nuaim AR Prevalence of glucose intolerance in urban and rural communities in Saudi Arabia Diabetic Medicine 1997 14 7 595 602 9223399 10.1002/(SICI)1096-9136(199707)14:7<595::AID-DIA377>3.0.CO;2-C

[B19] Bell R Bailey K Cresswell T Hawthorne G Critchley J Lewis Barned N Trends in prevalence and outcomes of pregnancy in women with pre existing type I and type II diabetes BJOG: An International Journal of Obstetrics & Gynaecology 2008 115 4 445 452 18271881 10.1111/j.1471-0528.2007.01644.x

[B20] Cheung NW McElduff A Ross GP Type 2 diabetes in pregnancy: a wolf in sheep's clothing Australian and New Zealand Journal of Obstetrics and Gynaecology 2005 45 6 479 483 16401211 10.1111/j.1479-828X.2005.00480.x

[B21] Feig DS Palda VA Type 2 diabetes in pregnancy: a growing concern Lancet 2002 359 9318 1690 1692 12020549 10.1016/S0140-6736(02)08599-9

[B22] Mathiesen ER Ringholm L Damm P Stillbirth in diabetic pregnancies Best Practice & Research Clinical Obstetrics & Gynaecology 2011 25 1 105 111 21256813 10.1016/j.bpobgyn.2010.11.001

[B23] Ganesh KS Unnikrishnan B Nagaraj K Jayaram S Determinants of Pre-eclampsia: A Case–control Study in a District Hospital in South India Indian Journal of Community Medicine: Official Publication of Indian Association of Preventive & Social Medicine 2010 35 4 502 21278871 10.4103/0970-0218.74360 PMC3026129

[B24] Shamsi U Hatcher J Shamsi A Zuberi N Qadri Z Saleem S A multicentre matched case control study of risk factors for Preeclampsia in healthy women in Pakistan BMC Women's Health 2010 10 1 14 20433699 10.1186/1472-6874-10-14 PMC2881882

[B25] Ray JG O'Brien TE Chan WS Preconception care and the risk of congenital anomalies in the offspring of women with diabetes mellitus: a meta analysis QJM 2001 94 8 435 11493721 10.1093/qjmed/94.8.435

[B26] Walkinshaw SA Pregnancy in women with pre-existing diabetes: management issues 2005 307 10.1016/j.siny.2005.04.004 15927547

[B27] Jensen DM Damm P Moelsted-Pedersen L Ovesen P Westergaard JG Moeller M Beck-Nielsen H Outcomes in type 1 diabetic pregnancies Diabetes Care 2004 27 12 2819 15562191 10.2337/diacare.27.12.2819

[B28] Ehrenberg HM Mercer BM Catalano PM The influence of obesity and diabetes on the prevalence of macrosomia American journal of obstetrics and gynecology 2004 191 3 964 15467573 10.1016/j.ajog.2004.05.052

[B29] Gómez HL Martínez ML Rodríguez ZM Clinical and Epidemiological Profile of Diabetes Mellitus in Pregnancy, Isle of Youth, 2008 MEDICC review 2011 13 1 29 21273956 10.37757/MR2011V13.N1.8

[B30] Weintrob N Karp M Hod M Short-and long-range complications in offspring of diabetic mothers Journal of Diabetes and its Complications 1996 10 5 294 301 8887019 10.1016/1056-8727(95)00080-1

[B31] Vitoratos N Vrachnis N Valsamakis G Panoulis K Creatsas G Perinatal mortality in diabetic pregnancy Annals of the New York Academy of Sciences 2010 1205 1 94 98 20840259 10.1111/j.1749-6632.2010.05670.x

[B32] Pregnancy outcomes in the Diabetes Control and Complications Trial Am J Obstet Gynecol 1996 174 4 1343 1353 8623868 10.1016/s0002-9378(96)70683-x

[B33] Boulot P Chabbert-Buffet N d'Ercole C Floriot M Fontaine P Fournier A Gillet JY Gin H Grandperret-Vauthier S Geudj AM French multicentric survey of outcome of pregnancy in women with pregestational diabetes Diabetes Care 2003 26 11 2990 14578228 10.2337/diacare.26.11.2990

[B34] Damm P Molsted-Pedersen L Significant decrease in congenital malformations in newborn infants of an unselected population of diabetic women Am J Obstet Gynecol 1989 161 5 1163 1167 2686445 10.1016/0002-9378(89)90656-x

[B35] Dicker D Feldberg D Samuel N Yeshaya A Karp M Goldman JA Spontaneous abortion in patients with insulin-dependent diabetes mellitus: the effect of preconceptional diabetic control Am J Obstet Gynecol 1988 158 5 1161 1164 3285689 10.1016/0002-9378(88)90245-1

[B36] Fuhrmann K Reiher H Semmler K Fischer F Fischer M Glockner E Prevention of congenital malformations in infants of insulin-dependent diabetic mothers Diabetes Care 1983 6 3 219 223 6347574 10.2337/diacare.6.3.219

[B37] FUHRMAN K REIHER li SEMMLER K E G The Effect of Intensified Conventional Insulin Therapy before and during Pregnancy on the Malformation Rate in Offspring of Diabetic.Mothers Clin Endocrinol 1984 83 2 5 10.1055/s-0029-1210327 6373320

[B38] Galindo A Garcia Burguillo A Azriel S De La Fuente P Artal R Outcome of fetuses in women with pregestational diabetes mellitus Journal of perinatal medicine 2006 34 4 323 332 16856824 10.1515/JPM.2006.062

[B39] Garcia-Patterson A Corcoy R Rigla M Caballero A Adelantado JM Altirriba O de Leiva A Does preconceptional counselling in diabetic women influence perinatal outcome? Ann Ist Super Sanita 1997 33 3 333 336 9542258

[B40] Goldman JA Dicker D Feldberg D Yeshaya A Samuel N Karp M Pregnancy outcome in patients with insulin-dependent diabetes mellitus with preconceptional diabetic control: a comparative study Am J Obstet Gynecol 1986 155 2 293 297 3740144 10.1016/0002-9378(86)90812-4

[B41] Jaffiol C Baccara MT Renard E Apostol DJ Lefebvre P Boulot P Daures JP Bringer J [Evaluation of the benefits brought by pregnancy planning in type 1 diabetes mellitus] Bull Acad Natl Med 2000 184 5 995 1007 discussion 1007-1008 11077720

[B42] Kitzmiller JL Gavin LA Gin GD Jovanovic-Peterson L Main EK Zigrang WD Preconception care of diabetes JAMA: The Journal of the American Medical Association 1991 265 6 731 1990188

[B43] McElvy SS Miodovnik M Rosenn B Khoury JC Siddiqi T Dignan PSJ Tsang RC A focused preconceptional and early pregnancy program in women with type 1 diabetes reduces perinatal mortality and malformation rates to general population levels Journal of Maternal-Fetal and Neonatal Medicine 2000 9 1 14 20 10.1002/(SICI)1520-6661(200001/02)9:1<14::AID-MFM5>3.0.CO;2-K 10757430

[B44] Mills JL Knopp RH Simpson JL Jovanovic-Peterson L Metzger BE Holmes LB Aarons JH Brown Z Reed GF Bieber FR Lack of relation of increased malformation rates in infants of diabetic mothers to glycemic control during organogenesis N Engl J Med 1988 318 11 671 676 3344018 10.1056/NEJM198803173181104

[B45] Rosenn B Miodovnik M Combs CA Khoury J Siddiqi TA Pre-conception management of insulin-dependent diabetes: improvement of pregnancy outcome Obstet Gynecol 1991 77 6 846 849 2030855

[B46] Rowe BR Rowbotham CJ Barnett AH Pre-conception counselling, birth weight, and congenital abnormalities in established and gestational diabetic pregnancy Diabetes research (Edinburgh, Scotland) 1987 6 1 33 3690954

[B47] Steel JM Johnstone FD Hepburn DA Smith AF Can prepregnancy care of diabetic women reduce the risk of abnormal babies? British Medical Journal 1990 301 6760 1070 2249069 10.1136/bmj.301.6760.1070 PMC1664221

[B48] Temple RC Aldridge VJ Murphy HR Prepregnancy care and pregnancy outcomes in women with type 1 diabetes Diabetes Care 2006 29 8 1744 16873774 10.2337/dc05-2265

[B49] Willhoite MB Bennert HW Palomaki GE Jr Zaremba MM Herman WH Williams JR Spear NH The impact of preconception counseling on pregnancy outcomes. The experience of the Maine Diabetes in Pregnancy Program Diabetes Care 1993 16 2 450 455 8432216 10.2337/diacare.16.2.450

[B50] Dunne FP Brydon P Smith T Essex M Nicholson H Dunn J Pre-conception diabetes care in insulin-dependent diabetes mellitus Qjm 1999 92 3 175 10326077 10.1093/qjmed/92.3.175

[B51] Garcia Ingelmo MT Herranz de la Morena L Martin Vaquero P Janez Furio M Grande Aragon C Pallardo Sanchez LF [Preconceptional control in diabetic women] Rev Clin Esp 1998 198 2 80 84 9580467

[B52] Jensen BM Kuhl C Molsted-Pedersen L Saurbrey N Fog-Pedersen J Preconceptional treatment with insulin infusion pumps in insulin-dependent diabetic women with particular reference to prevention of congenital malformations Acta Endocrinol Suppl (Copenh) 1986 277 81 85 3464152 10.1530/acta.0.111s0081

[B53] Murphy LE Gollenberg AL Louis GMB Kostyniak PJ Sundaram R Maternal serum preconception polychlorinated biphenyl concentrations and infant birth weight Environmental Health Perspectives 2010 118 2 297 20123616 10.1289/ehp.0901150 PMC2831933

[B54] Heller S Damm P Mersebach H Skjøth TV Kaaja R Hod M Durán-García S McCance D Mathiesen ER Hypoglycemia in Type 1 Diabetic Pregnancy Diabetes Care 2010 33 3 473 20007944 10.2337/dc09-1605 PMC2827491

[B55] Tripathi A Rankin J Aarvold J Chandler C Bell R Preconception Counseling in Women With Diabetes Diabetes Care 2010 33 3 586 20040652 10.2337/dc09-1585 PMC2827513

[B56] Temple RC Aldridge V Stanley K Murphy HR Glycaemic control throughout pregnancy and risk of pre-eclampsia in women with type I diabetes BJOG 2006 113 11 1329 1332 17004981 10.1111/j.1471-0528.2006.01071.x

[B57] Morrell MJ Guidelines for the care of women with epilepsy Neurology 1998 51 5 Supplement 4 S21 9818920 10.1212/wnl.51.5_suppl_4.s21

[B58] Stokes T Shaw EJ Juarez-Garcia A Camosso-Stefinovic J Baker R Clinical guidelines and evidence review for the epilepsies: diagnosis and management in adults and children in primary and secondary care London: Royal College of General Practitioners 2004

[B59] Barrett C Richens A Epilepsy and pregnancy: Report of an Epilepsy Research Foundation Workshop Epilepsy research 2003 52 3 147 12536051 10.1016/s0920-1211(02)00237-1

[B60] Adab N Kini U Vinten J Ayres J Baker G Clayton-Smith J Coyle H Fryer A Gorry J Gregg J The longer term outcome of children born to mothers with epilepsy Journal of Neurology, Neurosurgery & Psychiatry 2004 75 11 1575 10.1136/jnnp.2003.029132 PMC1738809 15491979

[B61] Adab N Tudur Smith C Vinten J Williamson PR Winterbottem JJ Common antiepileptic drugs in pregnancy in women with epilepsy 2004 1 status and date: Edited (no change to conclusions) 10.1002/14651858.CD004848 15266543

[B62] Tomson T Perucca E Battino D Navigating toward fetal and maternal health: the challenge of treating epilepsy in pregnancy Epilepsia 2004 45 10 1171 1175 15461670 10.1111/j.0013-9580.2004.15104.x

[B63] Bardy AH Seizure frequency in epileptic women during pregnancy and the puerperium: results of the prospective Helsinki Study Epilepsy, pregnancy, and the child 1982 27 31

[B64] Klein P Herzog AG Hormonal effects on epilepsy in women Epilepsia 1998 39 S9 S16 9915615 10.1111/j.1528-1157.1998.tb02602.x

[B65] Guberman A Hormonal contraception and epilepsy Neurology 1999 53 4 38 40 10487513

[B66] Vajda FJ O'Brien TJ Hitchcock A Graham J Lander C The Australian registry of anti-epileptic drugs in pregnancy: experience after 30 months Journal of Clinical Neuroscience 2003 10 5 543 549 12948456 10.1016/s0967-5868(03)00158-9

[B67] Wide K Winbladh B Källén B Major malformations in infants exposed to antiepileptic drugs in utero, with emphasis on carbamazepine and valproic acid: a nation-wide, population-based register study Acta Paediatrica 2004 93 2 174 176 15046269 10.1080/08035250310021118

[B68] Betts T Fox C Proactive pre-conception counselling for women with epilepsy--is it effective? Seizure 1999 8 6 322 327 10512771 10.1053/seiz.1999.0325

[B69] Lee PJ Pregnancy issues in inherited metabolic disorders Journal of inherited metabolic disease 2006 29 2 311 316 16763893 10.1007/s10545-005-0252-1

[B70] Rouse B Azen C Effect of high maternal blood phenylalanine on offspring congenital anomalies and developmental outcome at ages 4 and 6 years: the importance of strict dietary control preconception and throughout pregnancy The Journal of Pediatrics 2004 144 2 235 239 14760268 10.1016/j.jpeds.2003.10.062

[B71] Winterbottom JB Smyth RM Jacoby A Baker GA Preconception counselling for women with epilepsy to reduce adverse pregnancy outcome Cochrane database of systematic reviews (Online) 2008 3 10.1002/14651858.CD006645.pub2 18646164

[B72] Meador KJ Baker GA Browning N Clayton-Smith J Combs-Cantrell DT Cohen M Kalayjian LA Kanner A Liporace JD Pennell PB Cognitive function at 3 years of age after fetal exposure to antiepileptic drugs New England Journal of Medicine 2009 360 16 1597 19369666 10.1056/NEJMoa0803531 PMC2737185

[B73] NIH Phenylketonuria: screening and management. NIH Consensus Statement 2000 17 National Institutes of Health 11 27 11757784

[B74] ACOG committee opinion. Maternal phenylketonuria. Committee on Genetics Int J Gynaecol Obstet 2001 72 1 83 84 11203688

[B75] Koch R Hanley W Levy H Matalon R Rouse B Cruz FD Azen C Friedman EG A preliminary report of the collaborative study of maternal phenylketonuria in the United States and Canada Journal of inherited metabolic disease 1990 13 4 641 650 2122127 10.1007/BF01799519

[B76] Maillot F Lilburn M Baudin J Morley DW Lee PJ Factors influencing outcomes in the offspring of mothers with phenylketonuria during pregnancy: the importance of variation in maternal blood phenylalanine American Journal of Clinical Nutrition 2008 88 3 700 18779286 10.1093/ajcn/88.3.700

[B77] Germain S Nelson-Piercy C Lupus nephritis and renal disease in pregnancy Lupus 2006 15 3 148 16634368 10.1191/0961203306lu2281rr

[B78] Güttler F Lou H Andresen J Kok K Mikkelsen I Nielsen KB Nielsen JB Cognitive development in offspring of untreated and preconceptionally treated maternal phenylketonuria Journal of inherited metabolic disease 1990 13 4 665 671 2122130 10.1007/BF01799522

[B79] Drogari E Beasley M Smith I Lloyd JK TIMING OF STRICT DIET IN RELATION TO FETAL DAMAGE IN MATERNAL PHENYLKETONURIA* 1: An International Collaborative Study by the MRC/DHSS Phenylketonuria Register The Lancet 1987 330 8565 927 930 10.1016/s0140-6736(87)91418-8 2889860

[B80] Smith I Glossop J Beasley M Fetal damage due to maternal phenylketonuria: effects of dietary treatment and maternal phenylalanine concentrations around the time of conception Journal of inherited metabolic disease 1990 13 4 651 657 2122128 10.1007/BF01799520

[B81] Leung AS Millar LK Koonings PP Montoro M Mestman JH Perinatal outcome in hypothyroid pregnancies Obstetrics & Gynecology 1993 81 3 349 8437784

[B82] Sahu MT Das V Mittal S Agarwal A Sahu M Overt and subclinical thyroid dysfunction among Indian pregnant women and its effect on maternal and fetal outcome Archives of Gynecology and Obstetrics 2010 281 2 215 220 19437026 10.1007/s00404-009-1105-1

[B83] Davis LE Leveno KJ Cunningham FG Hypothyroidism complicating pregnancy Obstetrics & Gynecology 1988 72 1 108 3380497

[B84] Wasserstrum N Ananla CA Perinatal consequences of maternal hypothyroidism in early pregnancy and inadequate replacement Clinical endocrinology 1995 42 4 353 358 7750188 10.1111/j.1365-2265.1995.tb02642.x

[B85] Haddow JE Palomaki GE Allan WC Williams JR Knight GJ Gagnon J O'Heir CE Mitchell ML Hermos RJ Waisbren SE Maternal thyroid deficiency during pregnancy and subsequent neuropsychological development of the child 1999 341 549 555 10.1056/NEJM199908193410801 10451459

[B86] Glinoer D Riahi M Grun JP Kinthaert J Risk of subclinical hypothyroidism in pregnant women with asymptomatic autoimmune thyroid disorders Journal of Clinical Endocrinology & Metabolism 1994 79 1 197 8027226 10.1210/jcem.79.1.8027226

[B87] Iijima T Tada H Hidaka Y Mitsuda N Murata Y Amino N Effects of autoantibodies on the course of pregnancy and fetal growth Obstetrics & Gynecology 1997 90 3 364 9277645 10.1016/s0029-7844(97)00283-4

[B88] Negro R Formoso G Mangieri T Pezzarossa A Dazzi D Hassan H Levothyroxine treatment in euthyroid pregnant women with autoimmune thyroid disease: effects on obstetrical complications Journal of Clinical Endocrinology & Metabolism 2006 91 7 2587 16621910 10.1210/jc.2005-1603

[B89] Ghafoor F Mansoor M Malik T Malik MS Khan AU Edwards R Akhtar W Role of thyroid peroxidase antibodies in the outcome of pregnancy Journal of the College of Physicians and Surgeons--Pakistan: JCPSP 2006 16 7 468 16827958

[B90] Momotani N Ito K Treatment of pregnant patients with Basedow's disease Experimental and clinical endocrinology 1991 97 2-3 268 274 1680727 10.1055/s-0029-1211077

[B91] Mestman JH Parathyroid disorders of pregnancy 1998 Elsevier 485 496 10.1016/s0146-0005(98)80028-1 9880118

[B92] Mestman JH Hyperthyroidism in pregnancy Endocrinology &Metabolism Clinics of North America 1998 27 1 127 149 9534033 10.1016/s0889-8529(05)70303-0

[B93] Earl R Crowther CA Middleton P Interventions for preventing and treating hyperthyroidism in pregnancy Cochrane database of systematic reviews (Online) 2010 9 10.1002/14651858.CD008633.pub2 PMC4175534 20824882

[B94] Wing DA Millar LK Koonings PP Montoro MN Mestman JH A comparison of propylthiouracil versus methimazole in the treatment of hyperthyroidism in pregnancy American journal of obstetrics and gynecology 1994 170 1 Pt 1 90 8296851 10.1016/s0002-9378(94)70390-6

[B95] Browne ML Rasmussen SA Hoyt AT Waller DK Druschel CM Caton AR Canfield MA Lin AE Carmichael SL Romitti PA Maternal thyroid disease, thyroid medication use, and selected birth defects in the National Birth Defects Prevention Study Birth Defects Research Part A: Clinical and Molecular Teratology 2009 85 7 621 628 10.1002/bdra.20573 PMC6034621 19215015

[B96] Rotondi M Mazziotti G Sorvillo F Piscopo M Cioffi M Amato G Carella C Effects of increased thyroxine dosage pre-conception on thyroid function during early pregnancy European Journal of Endocrinology 2004 151 6 695 15588235 10.1530/eje.0.1510695

[B97] Vaquero E De Carolis C Valensise H Romanini C Lazzarin N Moretti C Mild Thyroid Abnormalities and Recurrent Spontaneous Abortion: Diagnostic and Therapeutical Approach1 American Journal of Reproductive Immunology 2000 43 4 204 208 10836249 10.1111/j.8755-8920.2000.430404.x

[B98] Petri M Hopkins Lupus Pregnancy Center: 1987 to 1996 Rheumatic diseases clinics of North America 1997 23 1 1 9031371 10.1016/s0889-857x(05)70311-2

[B99] Petri M Allbritton J Fetal outcome of lupus pregnancy: a retrospective case-control study of the Hopkins Lupus Cohort Obstetrical & gynecological survey 1993 48 11 717 8496859

[B100] Surita FGC Parpinelli MÂ Yonehara E Krupa F Cecatti JG Systemic lupus erythematosus and pregnancy: clinical evolution, maternal and perinatal outcomes and placental findings Sao Paulo Medical Journal 2007 125 91 95 17625706 10.1590/S1516-31802007000200005 PMC11014693

[B101] Petri M Systemic lupus erythematosus and pregnancy Rheumatic diseases clinics of North America 1994 20 1 87 8153405 PMC5288842

[B102] Huong D Wechsler B Vauthier-Brouzes D Beaufils H Lefebvre G Piette JC Pregnancy in past or present lupus nephritis: a study of 32 pregnancies from a single centre British Medical Journal 2001 60 6 599 10.1136/ard.60.6.599 PMC1753674 11350849

[B103] Loizou S Byron MA Englert HJ David J Hughes GRV Walport MJ Association of quantitative anticardiolipin antibody levels with fetal loss and time of loss in systemic lupus erythematosus Qjm 1988 68 1 525 3252304

[B104] Mintz G Niz J Gutierrez G Garcia-Alonso A Karchmer S Prospective study of pregnancy in systemic lupus erythematosus. Results of a multidisciplinary approach J Rheumatol 1986 13 4 732 739 3772921

[B105] Lockshin MD Reinitz E Druzin ML Murrman M Estes D Lupus pregnancy. Case-control prospective study demonstrating absence of lupus exacerbation during or after pregnancy Am J Med 1984 77 5 893 898 6496544 10.1016/0002-9343(84)90538-2

[B106] Nossent HC Swaak TJG Systemic lupus erythematosus. VI, Analysis of the interrelationship with pregnancy Journal of rheumatology 1990 17 6 771 776 2388198

[B107] Clark CA Spitzer KA Nadler JN Laskin CA Preterm deliveries in women with systemic lupus erythematosus The Journal of Rheumatology 2003 30 10 2127 14528505

[B108] Georgiou PE Politi EN Katsimbri P Sakka V Drosos AA Outcome of lupus pregnancy: a controlled study Rheumatology 2000 39 9 1014 10986308 10.1093/rheumatology/39.9.1014

[B109] Cortes-Hernandez J Ordi-Ros J Paredes F Casellas M Castillo F Vilardell-Tarres M Clinical predictors of fetal and maternal outcome in systemic lupus erythematosus: a prospective study of 103 pregnancies Rheumatology 2002 41 6 643 12048290 10.1093/rheumatology/41.6.643

[B110] Lima F Buchanan NMM Khamashta MA Kerslake S Hughes GRV Obstetric outcome in systemic lupus erythematosus 1995 Elsevier 184 192 10.1016/s0049-0172(95)80030-1 8650588

[B111] Sittiwangkul S Louthrenoo W Vithayasai P Sukitawut W Pregnancy outcome in Thai patients with systemic lupus erythematosus Asian Pacific journal of allergy and immunology/launched by the Allergy and Immunology Society of Thailand 1999 17 2 77 10466542

[B112] Ruiz-Irastorza G Lima F Alves J Khamashta MA Simpson J Hughes GRV Buchanan NMM Increased rate of lupus flare during pregnancy and the puerperium: a prospective study of 78 pregnancies Rheumatology 1996 35 2 133 10.1093/rheumatology/35.2.133 8612024

[B113] Urowitz MB Gladman DD Farewell VT Stewart J McDonald J Lupus and pregnancy studies Arthritis & Rheumatism 1993 36 10 1392 1397 8216399 10.1002/art.1780361011

[B114] Skomsvoll JF Aasarød K Salvesen KA Hoff M Wallenius M Rødevand E Koksvik HS Gilboe IM Nossent HC Systemic lupus erythematosus and pregnancy Tidsskrift for den Norske lægeforening: tidsskrift for praktisk medicin, ny række 2007 127 6 725 17363983

[B115] Daskalakis GJ Kontessis PS Papageorgiou IS Paraskevopoulos AP Digenis GE Karaiskakis PT Antsaklis AJ Zerefos NS Lupus Nephritis and Pregnancy Hypertension in Pregnancy 1998 17 1 23 30

[B116] Pajor A Pozsonyi T Nékám K Bakos L Haraszti L Paulin F Systemic lupus erythematosus and pregnancy (effect of pre-conception hematologic disorders on fetal outcome) Orvosi hetilap 1998 139 8 415 9524424

[B117] Carmona F Font J Cervera R Muñoz F Cararach V Balasch J Obstetrical outcome of pregnancy in patients with systemic lupus erythematosus. A study of 60 cases European Journal of Obstetrics & Gynecology and Reproductive Biology 1999 83 2 137 142 10391522 10.1016/s0301-2115(98)00312-1

[B118] Jungers P Dougados M Pelissier C Kuttenn F Tron F Lesavre P Bach JF Lupus nephropathy and pregnancy: report of 104 cases in 36 patients Archives of Internal Medicine 1982 142 4 771 7073417 10.1001/archinte.142.4.771

[B119] Houser MT Fish AJ Tagatz GE Williams PP Michael AF Pregnancy and systemic lupus erythematosus American journal of obstetrics and gynecology 1980 138 4 409 7424997 10.1016/0002-9378(80)90138-6

[B120] Hayslett JP Lynn RI Effect of pregnancy in patients with lupus nephropathy Kidney Int 1980 18 2 207 220 7441988 10.1038/ki.1980.129

[B121] Cavallasca JA Laborde HA Ruda-Vega H Nasswetter GG Maternal and fetal outcomes of 72 pregnancies in Argentine patients with systemic lupus erythematosus (SLE) Clinical rheumatology 2008 27 1 41 46 17516127 10.1007/s10067-007-0649-3

[B122] Bobrie G Liote F Houillier P Grünfeld JP Jungers P Pregnancy in lupus nephritis and related disorders American journal of kidney diseases: the official journal of the National Kidney Foundation 1987 9 4 339 3107375 10.1016/s0272-6386(87)80133-6

[B123] Chandran V Aggarwal A Misra R Active disease during pregnancy is associated with poor foetal outcome in Indian patients with systemic lupus erythematosus Rheumatology international 2005 26 2 152 156 15627198 10.1007/s00296-004-0540-3

[B124] Lipscomb KJ Clayton Smith J Clarke B Donnai P Harris R Outcome of pregnancy in women with Marfan's syndrome BJOG: An International Journal of Obstetrics & Gynaecology 1997 104 2 201 206 10.1111/j.1471-0528.1997.tb11045.x 9070139

[B125] Carmona F Font J Moga I Làzaro I Cervera R Pac V Balasch J Class III–IV proliferative lupus nephritis and pregnancy: a study of 42 cases American Journal of Reproductive Immunology 2005 53 4 182 188 15760379 10.1111/j.1600-0897.2005.00263.x

[B126] Imbasciati E Tincani A Gregorini G Doria A Moroni G Cabiddu G Marcelli D Pregnancy in women with pre-existing lupus nephritis: predictors of fetal and maternal outcome Nephrology Dialysis Transplantation 2009 24 2 519 10.1093/ndt/gfn348 18565977

[B127] Podjanee Phadungkiatwattana P Sirivatanapa P Tongsong T Outcomes of pregnancies complicated by systemic lupus erythematosus (SLE) JOURNAL-MEDICAL ASSOCIATION OF THAILAND 2007 90 10 1981 18041412

[B128] Wagner SJ Craici I Reed D Norby S Bailey K Wiste HJ Wood CM Moder KG Liang KP Liang KV Maternal and foetal outcomes in pregnant patients with active lupus nephritis Lupus 2009 18 4 342 19276302 10.1177/0961203308097575 PMC2724676

[B129] Smyth A Oliveira GHM Lahr BD Bailey KR Norby SM Garovic VD A Systematic Review and Meta-Analysis of Pregnancy Outcomes in Patients with Systemic Lupus Erythematosus and Lupus Nephritis Clinical Journal of the American Society of Nephrology 2010 10.2215/CJN.00240110 PMC3001786 20688887

[B130] Caton AR Bell EM Druschel CM Werler MM Mitchell AA Browne ML McNutt LA Romitti PA Olney RS Correa A Maternal hypertension, antihypertensive medication use, and the risk of severe hypospadias Birth Defects Research Part A: Clinical and Molecular Teratology 2008 82 1 34 40 10.1002/bdra.20415 18022875

[B131] Romundstad PR Smith GD Nilsen TIL Vatten LJ Associations of Prepregnancy Cardiovascular Risk Factors With the Offspring's Birth Weight Obstetrical & Gynecological Survey 2008 63 4 214

[B132] Romundstad PR Smith GD Nilsen TIL Vatten LJ Associations of prepregnancy cardiovascular risk factors with the offspring's birth weight American journal of epidemiology 2007 166 12 1359 17978410 10.1093/aje/kwm272

[B133] Magnussen EB Vatten LJ Lund-Nilsen TI Salvesen KÅ Smith GD Romundstad PR Prepregnancy cardiovascular risk factors as predictors of pre-eclampsia: population based cohort study BMJ 2007 335 7627 978 17975256 10.1136/bmj.39366.416817.BE PMC2072028

[B134] Hameed AB Sklansky MS Pregnancy: maternal and fetal heart disease Current Problems in Cardiology 2007 32 8 419 494 17643825 10.1016/j.cpcardiol.2007.04.004

[B135] Kircher S Schatz M Long L Variables affecting asthma course during pregnancy Annals of Allergy, Asthma & Immunology 2002 89 5 463 466 10.1016/S1081-1206(10)62082-0 12452203

[B136] Demissie K Breckenridge MB Rhoads GG Infant and maternal outcomes in the pregnancies of asthmatic women American journal of respiratory and critical care medicine 1998 158 4 1091 9769265 10.1164/ajrccm.158.4.9802053

[B137] Liu SL Wen SW Demissie K Marcoux S Kramer MS Abouleish A Nordborg C Nordborg E Petursdottir V Powell CVE Maternal asthma and pregnancy outcomes: a retrospective cohort studydiabetes 2001 24 2 411 412 10.1067/mob.2001.108073 11174486

[B138] Schatz M The efficacy and safety of asthma medications during pregnancy 2001 Elsevier 145 152 10.1053/sper.2001.24569 11453610

[B139] Schatz M Zeiger RS Harden K Hoffman CC Chilingar L Petitti D The safety of asthma and allergy medications during pregnancy Journal of allergy and clinical immunology 1997 100 3 301 306 9314340 10.1016/s0091-6749(97)70241-0

[B140] Lin S Munsie JPW Herdt-Losavio ML Bell E Druschel C Romitti PA Olney R Maternal asthma medication use and the risk of gastroschisis American journal of epidemiology 2008 168 1 73 18436535 10.1093/aje/kwn098 PMC6067822

[B141] Jones DC Hayslett JP Outcome of pregnancy in women with moderate or severe renal insufficiency The New England journal of medicine 1996 335 4 226 232 8657238 10.1056/NEJM199607253350402

[B142] Holley JL Bernardini J Quadri KHM Greenberg A Laifer SA Pregnancy outcomes in a prospective matched control study of pregnancy and renal disease Obstetrical & Gynecological Survey 1996 51 10 581

[B143] Jungers P Chauveau D Choukroun G Moynot A Skhiri H Houillier P Forget D Grünfeld JP Pregnancy in women with impaired renal function Clinical nephrology 1997 47 5 281 288 9181274

[B144] Aromaa M Rautava P Helenius H Sillanpää ML Prepregnancy Headache and the Well being of Mother and Newborn Headache: The Journal of Head and Face Pain 1996 36 7 409 415 10.1046/j.1526-4610.1996.3607409.x 8783471

[B145] Vukusic S Hutchinson M Hours M Moreau T Cortinovis Tourniaire P Adeleine P Confavreux C Pregnancy and multiple sclerosis (the PRIMS study): clinical predictors of post partum relapse Brain 2004 127 6 1353 15130950 10.1093/brain/awh152

[B146] Matt DW Borzelleca JF Toxic effects on the female reproductive system during pregnancy, parturition, and lactation Reproductive Toxicology 1995 1757795328

[B147] Women’s Health United States Department of Health and Human Services, Health Research Services Administration, Maternal and Child Health Bureau 2004 Rockville, USA: Women’s Health USA

[B148] Bitsko RH Reefhuis J Louik C Werler M Feldkamp ML Waller DK Frias J Honein MA Periconceptional use of weight loss products including ephedra and the association with birth defects Birth Defects Research Part A: Clinical and Molecular Teratology 2008 82 8 553 562 10.1002/bdra.20472 18553492

[B149] Ahn HK Choi JS Han JY Kim MH Chung JH Ryu HM Kim MY Yang JH Koong MK Nava-Ocampo AA Pregnancy outcome after exposure to oral contraceptives during the periconceptional period Human & experimental toxicology 2008 27 4 307 18684801 10.1177/0960327108092290

[B150] Thadhani R Stampfer MJ Chasan-Taber L Willett WC Curhan GC A prospective study of pregravid oral contraceptive use and risk of hypertensive disorders of pregnancy Contraception 1999 60 3 145 150 10640157 10.1016/s0010-7824(99)00079-7

[B151] Martínez Frías ML Bermejo E Rodríguez Pinilla E Frias JL Exstrophy of the cloaca and exstrophy of the bladder: two different expressions of a primary developmental field defect American Journal of Medical Genetics 2001 99 4 261 269 11251990 10.1002/ajmg.1210

[B152] Werler MM Bosco JLF Shapira SK Maternal vasoactive exposures, amniotic bands, and terminal transverse limb defects Birth Defects Research Part A: Clinical and Molecular Teratology 2009 85 1 52 57 10.1002/bdra.20524 PMC2741326 19067400

[B153] Kothari A Girling J Hypothyroidism in pregnancy: pre pregnancy thyroid status influences gestational thyroxine requirements BJOG: An International Journal of Obstetrics & Gynaecology 2008 115 13 1704 1708 18947343 10.1111/j.1471-0528.2008.01901.x

[B154] Krapels IPC Zielhuis GA Vroom F de Jong van den Berg L Kuijpers Jagtman AM van der Molen ABM Steegers Theunissen RPM Periconceptional health and lifestyle factors of both parents affect the risk of live born children with orofacial clefts Birth Defects Research Part A: Clinical and Molecular Teratology 2006 76 8 613 620 10.1002/bdra.20285 16955502

[B155] Ross DS Jones JL Lynch MF Toxoplasmosis, cytomegalovirus, listeriosis, and preconception care Maternal and Child Health Journal 2006 10 189 193 10.1007/s10995-006-0092-0 PMC1592156 16752091

[B156] Gavin NI Gaynes BN Lohr KN Meltzer-Brody S Gartlehner G Swinson T Perinatal depression: a systematic review of prevalence and incidence Obstetrics & Gynecology 2005 106 5 Part 1 1071 16260528 10.1097/01.AOG.0000183597.31630.db

[B157] Ferri CP Mitsuhiro SS Barros M Chalem E Guinsburg R Patel V Prince M Laranjeira R The impact of maternal experience of violence and common mental disorders on neonatal outcomes: a survey of adolescent mothers in Sao Paulo, Brazil BMC Public Health 2007 7 1 209 17705835 10.1186/1471-2458-7-209 PMC2018717

[B158] Dayan J Creveuil C Marks MN Conroy S Herlicoviez M Dreyfus M Tordjman S Prenatal depression, prenatal anxiety, and spontaneous preterm birth: a prospective cohort study among women with early and regular care Psychosomatic Medicine 2006 68 6 938 17079701 10.1097/01.psy.0000244025.20549.bd

[B159] Neggers Y Goldenberg R Cliver S Hauth J The relationship between psychosocial profile, health practices, and pregnancy outcomes Acta obstetricia et gynecologica Scandinavica 2006 85 3 277 285 16553174 10.1080/00016340600566121

[B160] Orr B Douce G Baillie S Parton R Coote J Adjuvant effects of adenylate cyclase toxin of Bordetella pertussis after intranasal immunisation of mice Vaccine 2007 25 1 64 71 16916566 10.1016/j.vaccine.2006.07.019

[B161] Bonari L Bennett H Einarson A Koren G Risks of untreated depression during pregnancy Canadian Family Physician 2004 50 1 37 14761100 PMC2214485

[B162] Field T Diego M Hernandez-Reif M Prenatal depression effects on the fetus and newborn: a review Infant Behavior and Development 2006 29 3 445 455 17138297 10.1016/j.infbeh.2006.03.003

[B163] Oberlander TF Warburton W Misri S Aghajanian J Hertzman C Neonatal outcomes after prenatal exposure to selective serotonin reuptake inhibitor antidepressants and maternal depression using population-based linked health data Archives of general psychiatry 2006 63 8 898 906 16894066 10.1001/archpsyc.63.8.898

[B164] Suri R Altshuler L Hellemann G Burt VK Aquino A Mintz J Effects of antenatal depression and antidepressant treatment on gestational age at birth and risk of preterm birth American Journal of Psychiatry 2007 164 8 1206 17671283 10.1176/appi.ajp.2007.06071172

[B165] Källén B Neonate characteristics after maternal use of antidepressants in late pregnancy Archives of pediatrics & adolescent medicine 2004 158 4 312 15066868 10.1001/archpedi.158.4.312

[B166] Hendrick V Treatment of postnatal depression BMJ 2003 327 7422 1003 14593008 10.1136/bmj.327.7422.1003 PMC261646

[B167] Curtis JR Engelberg RA Wenrich MD Au DH Communication about palliative care for patients with chronic obstructive pulmonary disease Journal of palliative care 2005 21 3 157 16334970

[B168] Tiet QQ Mausbach B Treatments for patients with dual diagnosis: a review Alcoholism: Clinical and Experimental Research 2007 31 4 513 536 10.1111/j.1530-0277.2007.00336.x 17374031

[B169] Krishnan V Bryant HU MacDougald OA Regulation of bone mass by Wnt signaling Journal of Clinical Investigation 2006 116 5 1202 16670761 10.1172/JCI28551 PMC1451219

[B170] Viguera AC Cohen LS Bouffard S Whitfield TH Baldessarini RJ Reproductive decisions by women with bipolar disorder after prepregnancy psychiatric consultation American Journal of Psychiatry 2002 159 12 2102 12450965 10.1176/appi.ajp.159.12.2102

[B171] Gavin AR Chae DH Mustillo S Kiefe CI Prepregnancy depressive mood and preterm birth in black and white women: Findings from the CARDIA study Journal of Women's Health 2009 18 6 803 10.1089/jwh.2008.0984 PMC2851123 19445645

[B172] Jonsson U Bohman H Hjern A von Knorring L Paaren A Olsson G von Knorring AL Intimate relationships and childbearing after adolescent depression: a population-based 15 year follow-up study Social Psychiatry and Psychiatric Epidemiology 2010 1 11 10.1007/s00127-010-0238-7 20512560

[B173] Silverman ME Loudon H Antenatal reports of pre-pregnancy abuse is associated with symptoms of depression in the postpartum period Archives of Women's Mental Health 2010 1 5 10.1007/s00737-010-0161-7 20386940

[B174] Harlow BL Vitonis AF Sparen P Cnattingius S Joffe H Hultman CM Incidence of hospitalization for postpartum psychotic and bipolar episodes in women with and without prior prepregnancy or prenatal psychiatric hospitalizations Archives of General Psychiatry 2007 64 1 42 17199053 10.1001/archpsyc.64.1.42

[B175] Tripathy P Nair N Barnett S Mahapatra R Borghi J Rath S Gope R Mahto D Sinha R Effect of a participatory intervention with women's groups on birth outcomes and maternal depression in Jharkhand and Orissa, India: a cluster-randomised controlled trial The Lancet 2010 375 9721 1182 1192 10.1016/S0140-6736(09)62042-0 20207411

[B176] Hirani SS Karmaliani R McFarlane J Asad N Madhani F testing a community derived intervention to promote women’s health: preliminary results of a 3-arm randomized controlled trial in Karachi, Pakistan 2010

[B177] Rychtarik RG McGillicuddy NB Coping skills training and 12-step facilitation for women whose partner has alcoholism: effects on depression, the partner's drinking, and partner physical violence Journal of Consulting and Clinical Psychology 2005 73 2 249 15796632 10.1037/0022-006X.73.2.249 PMC4652652

[B178] Miller LJ Finnerty M Sexuality, pregnancy, and childrearing among women with schizophrenia-spectrum disorders Families and Mental Health Treatment: A Compendium of Articles from Psychiatric Services and Hospital and Community Psychiatry 1996 45 10.1176/ps.47.5.502 8740491

[B179] Rudolph B Larson GL Sweeny S Hough EE Arorian K Hospitalized pregnant psychotic women: characteristics and treatment issues Psychiatric Services 1990 41 2 159 10.1176/ps.41.2.159 2078201

[B180] Miller LJ Psychotic denial of pregnancy: phenomenology and clinical management Psychiatric Services 1990 41 11 1233 10.1176/ps.41.11.1233 2249803

[B181] Seth P Raiji PT DiClemente RJ Wingood GM Rose E Psychological distress as a correlate of a biologically confirmed STI, risky sexual practices, self-efficacy and communication with male sex partners in African-American female adolescents Psychology health & medicine 2009 14 3 291 300 10.1080/13548500902730119 19444707

[B182] Viguera AC Nonacs R Cohen LS Tondo L Murray A Baldessarini RJ Risk of recurrence of bipolar disorder in pregnant and nonpregnant women after discontinuing lithium maintenance Am J Psychiatry 2000 157 2 179 184 10671384 10.1176/appi.ajp.157.2.179

[B183] Freeman MP Smith KW Freeman SA McElroy SL Kmetz GE Wright R Keck PE Jr The impact of reproductive events on the course of bipolar disorder in women J Clin Psychiatry 2002 63 4 284 287 12004800 10.4088/jcp.v63n0403

[B184] Li J Vestergaard M Obel C Christensen J Precht DH Lu M Olsen J A nationwide study on the risk of autism after prenatal stress exposure to maternal bereavement Pediatrics 2009 123 4 1102 1107 19336368 10.1542/peds.2008-1734

[B185] Li J Vestergaard M Obel C Precht DH Christensen J Lu M Olsen J Prenatal stress and epilepsy in later life: a nationwide follow-up study in Denmark Epilepsy Res 2008 81 1 52 57 18514486 10.1016/j.eplepsyres.2008.04.014

[B186] Li J Olsen J Obel C Christensen J Precht DH Vestergaard M Prenatal stress and risk of febrile seizures in children: a nationwide longitudinal study in Denmark J Autism Dev Disord 2009 39 7 1047 1052 19291382 10.1007/s10803-009-0717-4 PMC2694316

[B187] Li J Olsen J Vestergaard M Obel C Attention-deficit/hyperactivity disorder in the offspring following prenatal maternal bereavement: a nationwide follow-up study in Denmark Eur Child Adolesc Psychiatry 2010 19 10 747 753 20495989 10.1007/s00787-010-0113-9

[B188] Li J Olsen J Vestergaard M Obel C Baker JL Sorensen TI Prenatal stress exposure related to maternal bereavement and risk of childhood overweight PLoS One 2010 5 7 e11896 20689593 10.1371/journal.pone.0011896 PMC2912844

[B189] Hansen D Lou HC Olsen J Serious life events and congenital malformations: a national study with complete follow-up Lancet 2000 356 9233 875 880 11036891 10.1016/S0140-6736(00)02676-3

[B190] WHO R Preventing intimate partner and sexual violence against women Taking action and generating Evidence 2010 10.1136/ip.2010.029629 20921563

[B191] Coker AL Sanderson M Dong B Partner violence during pregnancy and risk of adverse pregnancy outcomes Paediatr Perinat Epidemiol 2004 18 4 260 269 15255879 10.1111/j.1365-3016.2004.00569.x

[B192] Janssen PA Holt VL Sugg NK Emanuel I Critchlow CM Henderson AD Intimate partner violence and adverse pregnancy outcomes: a population-based study Am J Obstet Gynecol 2003 188 5 1341 1347 12748509 10.1067/mob.2003.274

[B193] Alio AP Daley EM Nana PN Duan J Salihu HM Intimate partner violence and contraception use among women in Sub-Saharan Africa International journal of gynaecology and obstetrics: the official organ of the International Federation of Gynaecology and Obstetrics 2009 107 1 35 10.1016/j.ijgo.2009.05.002 19481751

[B194] Glander SS Moore ML Michielutte R Parsons LH The prevalence of domestic violence among women seeking abortion Obstet Gynecol 1998 91 6 1002 1006 9611013 10.1016/s0029-7844(98)00089-1

[B195] Hedin LW Janson PO Domestic violence during pregnancy. The prevalence of physical injuries, substance use, abortions and miscarriages Acta Obstet Gynecol Scand 2000 79 8 625 630 10949224 10.1080/j.1600-0412.2000.079008625.x

[B196] Salam MA Alim MA Noguchi T Spousal abuse against women and its consequences on reproductive health: a study in the urban slums in Bangladesh Maternal and Child Health Journal 2006 10 1 83 94 16362235 10.1007/s10995-005-0030-6

[B197] Beydoun HA Al-Sahab B Beydoun MA Tamim H Intimate Partner Violence as a Risk Factor for Postpartum Depression Among Canadian Women in the Maternity Experience Survey Annals of epidemiology 2010 20 8 575 583 20609336 10.1016/j.annepidem.2010.05.011 PMC4179881

[B198] Coker AL Weston R Creson DL Justice B Blakeney P PTSD symptoms among men and women survivors of intimate partner violence: The role of risk and protective factors Violence and victims 2005 20 6 625 643 16468442

[B199] Goodwin MM Gazmararian JA Johnson CH Gilbert BC Saltzman LE Pregnancy intendedness and physical abuse around the time of pregnancy: findings from the pregnancy risk assessment monitoring system, 1996-1997. PRAMS Working Group. Pregnancy Risk Assessment Monitoring System Matern Child Health J 2000 4 2 85 92 10994576 10.1023/a:1009566103493

[B200] Pallitto CC O'Campo P The Relationship between Intimate Partner Violence and Unintended Pregnancy: Analysis of a National Sample from Colombia International Family Planning Perspectives 2004 30 4 165 174 15590382 10.1363/3016504

[B201] Clark CJ Silverman J Khalaf IA Ra'ad BA Al Sha'ar ZA Al Ata AA Batieha A Intimate partner violence and interference with women's efforts to avoid pregnancy in Jordan Studies in Family Planning 2008 39 2 123 132 18678176 10.1111/j.1728-4465.2008.00159.x

[B202] Bauer HM Gibson P Hernandez M Kent C Klausner J Bolan G Intimate partner violence and high-risk sexual behaviors among female patients with sexually transmitted diseases Sexually transmitted diseases 2002 29 7 411 12170131 10.1097/00007435-200207000-00009

[B203] Stephenson R Koenig MA Ahmed S Domestic violence and symptoms of gynecologic morbidity among women in North India International Family Planning Perspectives 2006 32 4 201 208 17237017 10.1363/3220106

[B204] Seth P Raiford JL Robinson LSS Wingood GM DiClemente RJ Intimate partner violence and other partner-related factors: correlates of sexually transmissible infections and risky sexual behaviours among young adult African American women Sexual Health 2010 7 1 25 30 20152092 10.1071/SH08075

[B205] Bonomi AE Thompson RS Anderson M Reid RJ Carrell D Dimer JA Rivara FP Intimate partner violence and women's physical, mental, and social functioning Am J Prev Med 2006 30 6 458 466 16704938 10.1016/j.amepre.2006.01.015

[B206] Ramsay J Carter Y Davidson L Dunne D Eldridge S Hegarty K Rivas C Taft A Warburton A Feder G Advocacy interventions to reduce or eliminate violence and promote the physical and psychosocial well-being of women who experience intimate partner abuse 2009 4 status and date: Edited (no change to conclusions) 10.1002/14651858.CD005043.pub2 19588364

[B207] McFarlane J Parker B Soeken K Abuse during pregnancy: frequency, severity, perpetrator, and risk factors of homicide Public Health Nurs 1995 12 5 284 289 7479535 10.1111/j.1525-1446.1995.tb00150.x

[B208] Rodríguez M Valentine JM Son JB Muhammad M Intimate partner violence and barriers to mental health care for ethnically diverse populations of women Trauma, Violence, & Abuse 2009 10 4 358 10.1177/1524838009339756 PMC2761218 19638359

[B209] Forte T Cohen MM Du Mont J Hyman I Romans S Psychological and physical sequelae of intimate partner violence among women with limitations in their activities of daily living Archives of women's mental health 2005 8 4 248 256 10.1007/s00737-005-0093-9 16010449

[B210] Jewkes RK Dunkle K Nduna M Shai N Intimate partner violence, relationship power inequity, and incidence of HIV infection in young women in South Africa: a cohort study The Lancet 2010 376 9734 41 48 10.1016/S0140-6736(10)60548-X 20557928

[B211] Macy RJ Martin SL Kupper LL Casanueva C Guo S Partner Violence Among Women Before, During, and After Pregnancy: Multiple Opportunities for Intervention Women's Health Issues 2007 17 5 290 299 17659882 10.1016/j.whi.2007.03.006

[B212] Ellsberg M Jansen HAFM Heise L Watts CH Garcia-Moreno C Intimate partner violence and women's physical and mental health in the WHO multi-country study on women's health and domestic violence: an observational study The Lancet 2008 371 9619 1165 1172 10.1016/S0140-6736(08)60522-X 18395577

[B213] McCauley J Kern DE Kolodner K Dill L Schroeder AF DeChant HK Ryden J Bass EB Derogatis LR The “battering syndrome”: prevalence and clinical characteristics of domestic violence in primary care internal medicine practices Annals of internal medicine 1995 123 10 737 7574191 10.7326/0003-4819-123-10-199511150-00001

[B214] Saltzman LE Johnson CH Gilbert BC Goodwin MM Physical abuse around the time of pregnancy: an examination of prevalence and risk factors in 16 states Maternal and Child Health Journal 2003 7 1 31 43 12710798 10.1023/a:1022589501039

[B215] Raj A Liu R McCleary-Sills J Silverman JG South Asian victims of intimate partner violence more likely than non-victims to report sexual health concerns Journal of Immigrant Health 2005 7 2 85 91 15789160 10.1007/s10903-005-2641-9

[B216] Wupperman P Amble P Devine S Zonana H Fals-Stewart W Easton C Violence and Substance Use Among Female Partners of Men in Treatment for Intimate-Partner Violence Journal of the American Academy of Psychiatry and the Law Online 2009 37 1 75 19297637

[B217] Decker MR Seage GR Hemenway D Gupta J Raj A Silverman JG Intimate partner violence perpetration, standard and gendered STI/HIV risk behaviour, and STI/HIV diagnosis among a clinic-based sample of men Sexually transmitted infections 2009 85 7 555 19625287 10.1136/sti.2009.036368 PMC3623286

[B218] Gómez AM Speizer IS Beauvais H Sexual violence and reproductive health among youth in Port-au-Prince, Haiti Journal of Adolescent Health 2009 44 5 508 510 19380102 10.1016/j.jadohealth.2008.09.012 PMC3735616

[B219] Smedslund G Dalsbø TK Steiro AK Winsvold A Clench-Aas J Cognitive behavioural therapy for men who physically abuse their female partner (Review) 2011 10.1002/14651858.CD006048.pub2 PMC12047670 17636823

[B220] Decker MR Miller E Kapur NA Gupta J Raj A Silverman JG Intimate partner violence and sexually transmitted disease symptoms in a national sample of married Bangladeshi women International Journal of Gynecology & Obstetrics 2008 100 1 18 23 17904559 10.1016/j.ijgo.2007.06.045

[B221] McFarlane J Malecha A Watson K Gist J Batten E Hall I Smith S Intimate partner sexual assault against women: frequency, health consequences, and treatment outcomes Obstetrics & Gynecology 2005 105 1 99 15625149 10.1097/01.AOG.0000146641.98665.b6

[B222] Hurwitz EJH Gupta J Liu R Silverman JG Raj A Intimate partner violence associated with poor health outcomes in US South Asian women Journal of Immigrant and Minority Health 2006 8 3 251 261 16791535 10.1007/s10903-006-9330-1

[B223] Stein MB Kennedy C Major depressive and post-traumatic stress disorder comorbidity in female victims of intimate partner violence Journal of Affective Disorders 2001 66 2-3 133 138 11578665 10.1016/s0165-0327(00)00301-3

[B224] Hegarty K Gunn J Chondros P Small R Association between depression and abuse by partners of women attending general practice: descriptive, cross sectional survey British Medical Journal 2004 328 7440 621 15016694 10.1136/bmj.328.7440.621 PMC381136

[B225] Modie-Moroka T Intimate Partner Violence and Sexually Risky Behavior in Botswana: Implications for HIV Prevention Health Care for Women International 2009 30 3 230 231 19191117 10.1080/07399330802662036

[B226] Naimi TS Lipscomb LE Brewer RD Gilbert BC Binge drinking in the preconception period and the risk of unintended pregnancy: implications for women and their children Pediatrics 2003 111 5 1136 12728126

[B227] Martin SL Kilgallen B Tsui AO Maitra K Singh KK Kupper LL Sexual behaviors and reproductive health outcomes JAMA: The Journal of the American Medical Association 1999 282 20 1967 10.1001/jama.282.20.1967 10580466

[B228] Silverman JG Decker MR Reed E Raj A Intimate partner violence victimization prior to and during pregnancy among women residing in 26 US states: associations with maternal and neonatal health American journal of obstetrics and gynecology 2006 195 1 140 148 16813751 10.1016/j.ajog.2005.12.052

[B229] Ruiz-Pérez I Plazaola-Castaño J del Río-Lozano M Physical health consequences of intimate partner violence in Spanish women The European Journal of Public Health 2007 17 5 437 10.1093/eurpub/ckl280 17244672

[B230] Plichta SB Falik M Prevalence of violence and its implications for women's health Women's Health Issues 2001 11 3 244 258 11336864 10.1016/s1049-3867(01)00085-8

[B231] Ishida K Stupp P Melian M Serbanescu F Goodwin M Exploring the associations between intimate partner violence and women's mental health: Evidence from a population-based study in Paraguay Social Science & Medicine 2010 10.1016/j.socscimed.2010.08.007 20864237

[B232] Martin SL Rentz ED Chan RL Givens J Sanford CP Kupper LL Garrettson M Macy RJ Physical and Sexual Violence Among North Carolina Women:: Associations with Physical Health, Mental Health, and Functional Impairment Women's Health Issues 2008 18 2 130 140 18319149 10.1016/j.whi.2007.12.008

[B233] Coker AL Smith PH Bethea L King MR McKeown RE Physical health consequences of physical and psychological intimate partner violence Archives of family medicine 2000 9 5 451 10810951 10.1001/archfami.9.5.451

[B234] Ellsberg M Caldera T Herrera A Winkvist A Kullgren G Domestic violence and emotional distress among Nicaraguan women: Results from a population-based study American Psychologist 1999 54 1 30

[B235] Åsling Monemi K Tabassum Naved R Persson LÅ Violence against women and the risk of under five mortality: analysis of community based data from rural Bangladesh Acta Paediatrica 2008 97 2 226 232 18254912 10.1111/j.1651-2227.2007.00597.x

[B236] Raj A Santana MC La Marche A Amaro H Cranston K Silverman JG Perpetration of intimate partner violence associated with sexual risk behaviors among young adult men American journal of public health 2006 96 10 1873 16670216 10.2105/AJPH.2005.081554 PMC1586132

[B237] Dude A Intimate partner violence and increased lifetime risk of sexually transmitted infection among women in Ukraine Studies in Family Planning 2007 38 2 89 100 17642410 10.1111/j.1728-4465.2007.00120.x

[B238] Fanslow J Whitehead A Silva M Robinson E Contraceptive use and associations with intimate partner violence among a population based sample of New Zealand women Australian and New Zealand Journal of Obstetrics and Gynaecology 2008 48 1 83 89 18275577 10.1111/j.1479-828X.2007.00805.x

[B239] Dunkle KL Jewkes RK Nduna M Levin J Jama N Khuzwayo N Koss MP Duvvury N Perpetration of partner violence and HIV risk behaviour among young men in the rural Eastern Cape, South Africa Aids 2006 20 16 2107 17053357 10.1097/01.aids.0000247582.00826.52

[B240] Grisso JA Schwarz DF Hirschinger N Sammel M Brensinger C Santanna J Lowe RA Anderson E Shaw LM Bethel CA Violent injuries among women in an urban area New England journal of medicine 1999 341 25 1899 1905 10601510 10.1056/NEJM199912163412506

[B241] Chang JC Cluss PA Ranieri L Hawker L Buranosky R Dado D McNeil M Scholle SH Health care interventions for intimate partner violence: what women want Women's health issues: official publication of the Jacobs Institute of Women's Health 2005 15 1 21 10.1016/j.whi.2004.08.007 15661584

[B242] Riddell T Ford-Gilboe M Leipert B Strategies Used by Rural Women to Stop, Avoid, or Escape From Intimate Partner Violence Health Care for Women International 2009 30 1 134 159 19116826 10.1080/07399330802523774

[B243] Scott MC Easton CJ Racial differences in treatment effect among men in a substance abuse and domestic violence program Am J Drug Alcohol Abuse 2010 36 6 357 362 20936990 10.3109/00952990.2010.501131 PMC3666933

[B244] Babcock JC Green CE Robie C Does batterers' treatment work? A meta-analytic review of domestic violence treatment Clinical Psychology Review 2004 23 8 1023 1053 14729422 10.1016/j.cpr.2002.07.001

[B245] O'Leary KD Heyman RE Neidig PH Treatment of wife abuse: A comparison of gender-specific and conjoint approaches Behavior Therapy 1999 30 3 475 505

[B246] Markman HJ Renick MJ Floyd FJ Stanley SM Clements M Preventing marital distress through communication and conflict management training: A 4-and 5-year follow-up Journal of Consulting and Clinical Psychology 1993 61 1 70 8450110 10.1037//0022-006x.61.1.70

[B247] Simpson LE Atkins DC Gattis KS Christensen A Low-level relationship aggression and couple therapy outcomes Journal of Family Psychology 2008 22 1 102 18266537 10.1037/0893-3200.22.1.102

[B248] Stith SM Rosen H McCollum EE Thomsen CJ TREATING INTIMATE PARTNER VIOLENCE WITHIN INTACT COUPLE RELATIONSHIPS: OUTCOMES OF MULTI COUPLE VERSUS INDIVIDUAL COUPLE THERAPY Journal of Marital and Family Therapy 2004 30 3 305 318 15293649 10.1111/j.1752-0606.2004.tb01242.x

[B249] Labriola M Rempel M Davis RC Testing the effectiveness of batterer programs and judicial monitoring Final report (National Institute of Justice, Washington, DC) 2005

[B250] Davis RC Taylor BG Maxwell CD Victim Services R Does Batterer Treatment Reduce Violence? A Randomized Experiment in Brooklyn-Executive Summary Included 2000

[B251] Feder L Forde DR Test of the efficacy of court Mandated counseling for domestic violence offenders: The Broward Experiment, Executive Summary Washington, DC: National Institute of Justice 2000

[B252] Dunford FW The San Diego Navy Experiment: An assessment of interventions for men who assault their wives Journal of Consulting and Clinical Psychology 2000 68 3 468 10883563 10.1037//0022-006x.68.3.468

[B253] Saunders DG Feminist-cognitive-behavioral and process-psychodynamic treatments for men who batter: Interaction of abuser traits and treatment models Violence and victims 1996 11 4 393 414 9210279

[B254] Ruiz-Pérez I Plazaola-Castaño J Cáliz-Cáliz R Rodríguez-Calvo I García-Sánchez A Ferrer-González MÁ Guzmán-Úbeda M del Río-Lozano M López-Chicheri García I Risk factors for fibromyalgia: the role of violence against women Clinical rheumatology 2009 28 7 777 786 19277813 10.1007/s10067-009-1147-6

[B255] Miller E Breslau J Chung WJ Green JG McLaughlin KA Kessler RC Adverse childhood experiences and risk of physical violence in adolescent dating relationships Journal of epidemiology and community health 2011 10.1136/jech.2009.105429 PMC3686487 21321063

[B256] Wahabi HA Alzeidan RA Bawazeer GA Alansari LA Esmaeil SA Preconception care for diabetic women for improving maternal and fetal outcomes: a systematic review and meta-analysis BMC pregnancy and childbirth 2010 10 1 63 20946676 10.1186/1471-2393-10-63 PMC2972233

[B257] Crawford P Lee P Gender difference in management of epilepsy--what women are hearing Seizure 1999 8 3 135 139 10356368 10.1053/seiz.1999.0274

[B258] Sablock U Lindow SW Arnott PIE Masson EA Prepregnancy counselling for women with medical disorders Journal of Obstetrics and Gynaecology 2002 22 6 637 638 12554253 10.1080/0144361021000020439

