# Supplementary material for: Preconception care: screening and management of chronic disease and promoting psychological health
Source: Reprod Health. 2014 Sep 26;11(Suppl 3):S5. doi: 10.1186/1742-4755-11-S3-S5 (PMC4196564; doi:10.1186/1742-4755-11-S3-S5)
Supplement: Additional file 1 — Peer review reports. [file 1742-4755-11-S3-S5-S1.pdf]

Referee's comments to the authors– this sheet WILL be seen by the author(s) and published with the article

Title	**Preconception - Screening and management of chronic disease promoting psychological health**
Author(s)	Zohra S Lassi, Ayesha M Imam, Sohni V Dean, Zulfiqar A Bhutta
Referee's name	Aamer Imdad

When assessing the work, please consider the following points, where applicable:

1. Is the question posed by the authors new and well defined?
2. Are the methods appropriate and well described, and are sufficient details provided to replicate the work?
3. Are the data sound and well controlled?
4. Does the manuscript adhere to the relevant standards for reporting and data deposition?
5. Are the discussion and conclusions well balanced and adequately supported by the data?
6. Do the title and abstract accurately convey what has been found?
7. Is the writing acceptable?

Please make your report as constructive and detailed as possible in your comments so that authors have the opportunity to overcome any serious deficiencies that you find and please also divide your comments into the following categories:

- Major Compulsory Revisions (which the author must respond to before a decision on publication can be reached)
- Minor Essential Revisions (such as missing labels on figures, or the wrong use of a term, which the author can be trusted to correct)
- Discretionary Revisions (which are recommendations for improvement but which the author can choose to ignore)

Where possible please supply references to substantiate your comments.

When referring to the manuscript please provide specific page and paragraph citations where appropriate.

General comments:
This paper discusses very important issue of pre-conception care for women of reproductive with chronic diseases. It is however noticed that paper need significant improvement and lacks explanation of key aspect of a meta-analysis. Objectives of the review are not well defined. Methods are not adequately described and result section has a lot of redundant text that can be presented in form of tables and a separate discussion section (which this paper does not have). I have describe the feedback in more specific details below.

Major compulsory revisions:

1) **Title of the study do not reflect the contents/objectives of the study, I would suggest the title as "Pre-conception care for reproductive women with chronic disease and mental health issues and its effect on maternal and neonatal outcomes".**
2) **Abstract does not represent an accurate summary of the paper. Objectives of the papers are not clearly mentioned in the abstract. How come the conclusion of the paper in abstract is the exact statement given in the last paragraph of the introduction section?**
3) **Methods:**
    a) **What are the primary outcomes of the paper?**
    b) **How was meta-analysis conducted? How were categorical and continuous variables handled? How were summary estimates presented? What criteria were used to determine the statistical**

heterogeneity in the pooled estimates? What software was used to conduct the meta-analysis?

c) **Were there any *a-priori* hypotheses to conduct the subgroup and sensitivity analyses?**
d) **Was data double extracted? Did authors use any standardized sheet to extract the data?**
e) **How was risk of bias determined in the published studies?**
f) **What were the inclusion and exclusion criteria?**
g) **What constitutes the "Pre-conception" care?**
h) **Was "family planning" and "Pre-conception care" taken as synonymous?**
i) **What about women of reproductive age with: Pre-pregnancy Respiratory disease (like cystic fibrosis); Endocrine problems (like polycystic ovarian syndrome) and women with Cancers (especially, hematologic malignancies Leukemia and lymphomas, breast cancer and cervical cancer)? Why were these conditions not studied as part of this review?**

4) **Results:**
   a) **Provide a table for "characteristics of included studies" for each chronic disease. They can probably be included as web-tables**
   b) **Were there any studies that studied differential effect of Type 1 vs. type 2 diabetes on pregnancy outcomes?**
   c) **What pre-conception care was given in 21 studies included in meta-analysis for diabetes? Was it same in all the studies?**
   d) **It seems like that there was a lot of clinical heterogeneity among studies of pre-gestational diabetes in terms of study design, interventions and outcomes. Please comment on that.**
   e) **First sentence on Section of Phenylketonuria is an exact copy of the first sentence of abstract of reference 66.**
   f) **There is a lot of redundant text in result's section that can be reduced by making tables for "Characteristics of included studies".**
   g) **It was noticed that authors tend to describe the complications of a particular medical condition rather than "pre-conception care" especially towards the end of results section.**
   h) **Side effect of medications should be mentioned in their respective section.**
5) **Describe the limitations of the review.**
6) **What are the research gaps?**

Minor essential revisions:

1. **Abstract: Provide meaning of abbreviation used in Abstract: MNCH, CI, MD.**
2. **Introduction: Give reference for the claims made in last paragraph of introduction section.**
3. **Methods:**
   a) **What electronic databases were exactly searched? What was the search strategy?**
   b) **What "Standard quality criteria" was used to evaluate the quality of studies? Reference?**
   c) **How was Type 1 and type diabetes defined?**
   d) **What was the definition of Epilepsy?**

4. **Results:**
   a) **Flow diagram can further show the number of studies found for each aspect of the review (e.g. no of studies for diabetes out of 70.**
   b) **Figure 1 and 2 are not visible in the document sent to me.**
   c) **It is noticed that authors have referred results of previous reviews in result section on diabetes? Does this section include both discussion and results related to management of pre-gestational diabetes?**
   d) **Section on Epilepsy: What does this sentence mean "Most women with epilepsy have no change in seizure frequency during pregnancy but about 15-33% have more seizures during pregnancy"? I think more seizures mean increase frequency???**
   e) **"Many studies looking at the effect of preconception counseling in women with chronic disorders versus healthy women have also looked into women with epilepsy but there comparison group was not relevant to this review, hence these studies were not included." What was that comparison group?**

Referee's comments to the authors– this sheet WILL be seen by the author(s) and published with the article

Title	**Preconception - Screening and management of chronic disease promoting psychological health**
Author(s)	Zohra S Lassi, Ayesha M Imam, Sohni V Dean, Zulfiqar A Bhutta
Referee's name	Khadija Humayun

When assessing the work, please consider the following points, where applicable:

1. Is the question posed by the authors new and well defined?
2. Are the methods appropriate and well described, and are sufficient details provided to replicate the work?
3. Are the data sound and well controlled?
4. Does the manuscript adhere to the relevant standards for reporting and data deposition?
5. Are the discussion and conclusions well balanced and adequately supported by the data?
6. Do the title and abstract accurately convey what has been found?
7. Is the writing acceptable?

Please make your report as constructive and detailed as possible in your comments so that authors have the opportunity to overcome any serious deficiencies that you find and please also divide your comments into the following categories:

- Major Compulsory Revisions (which the author must respond to before a decision on publication can be reached)
- Minor Essential Revisions (such as missing labels on figures, or the wrong use of a term, which the author can be trusted to correct)
- Discretionary Revisions (which are recommendations for improvement but which the author can choose to ignore)

Where possible please supply references to substantiate your comments.

When referring to the manuscript please provide specific page and paragraph citations where appropriate.

General comments:

Major compulsory revisions:

Minor essential revisions:
The Title and Abstract do not do not accurately convey the findings of the authors.

The title " Preconception - Screening and management of chronic disease promoting Psychological health" seems to tell that screening and management of chronic disease promotes psychological health, whereas the psychological illness has been seen as a form of chronic disease so it needs to be rephrased.

The abstract does not summarize all the aspects the article deals with. In the abstract we get an idea about

(continue on the next sheet)

Continued:few issues only where as the review has much covered much more. Thus the abstract needs to be rewritten so that it is representative of the article.
There are few other minor things which have been highlighted in the text of the article and comment is inserted.

Referee's comments to the authors– this sheet WILL be seen by the author(s) and published with the article

Title	**Preconception - Screening and management of chronic disease promoting psychological health**
Author(s)	Zohra S Lassi, Ayesha M Imam, Sohni V Dean, Zulfiqar A Bhutta
Referee's name	Aamer Imdad

When assessing the work, please consider the following points, where applicable:

1. Is the question posed by the authors new and well defined?
2. Are the methods appropriate and well described, and are sufficient details provided to replicate the work?
3. Are the data sound and well controlled?
4. Does the manuscript adhere to the relevant standards for reporting and data deposition?
5. Are the discussion and conclusions well balanced and adequately supported by the data?
6. Do the title and abstract accurately convey what has been found?
7. Is the writing acceptable?

Please make your report as constructive and detailed as possible in your comments so that authors have the opportunity to overcome any serious deficiencies that you find and please also divide your comments into the following categories:

- Major Compulsory Revisions (which the author must respond to before a decision on publication can be reached)
- Minor Essential Revisions (such as missing labels on figures, or the wrong use of a term, which the author can be trusted to correct)
- Discretionary Revisions (which are recommendations for improvement but which the author can choose to ignore)

Where possible please supply references to substantiate your comments.

When referring to the manuscript please provide specific page and paragraph citations where appropriate.

General comments:
This paper discusses very important issue of pre-conception care for women of reproductive with chronic diseases. It is however noticed that paper need significant improvement and lacks explanation of key aspect of a meta-analysis. Objectives of the review are not well defined. Methods are not adequately described and result section has a lot of redundant text that can be presented in form of tables and a separate discussion section (which this paper does not have). I have describe the feedback in more specific details below.

Major compulsory revisions:

7) **Title of the study do not reflect the contents/objectives of the study, I would suggest the title as "Pre-conception care for reproductive women with chronic disease and mental health issues and its effect on maternal and neonatal outcomes".**

We have now changed the title. We actually missed the word "and" and with its addition, the title is now making sense. "Preconception Care- Screening and management of chronic disease and promoting psychological health"

8) **Abstract does not represent an accurate summary of the paper. Objectives of the papers are not clearly mentioned in the abstract. How come the conclusion of the paper in abstract is the exact statement given in the last paragraph of the introduction section?**
   Revisited and revised the abstract.

9) **Methods:**
   j) **What are the primary outcomes of the paper?**
      Added in fourth line of the first para under the heading of methods "…improved maternal, newborn and child health outcomes"
   k) **How was meta-analysis conducted? How were categorical and continuous variables handled? How were summary estimates presented? What criteria were used to determine the statistical heterogeneity in the pooled estimates? What software was used to conduct the meta-analysis?**
      Added in the second para under the heading of methods
   l) **Were there any *a-priori* hypotheses to conduct the subgroup and sensitivity analyses?**
      Added in the second para under the heading of methods in the last sentence.
   m) **Was data double extracted? Did authors use any standardized sheet to extract the data?**
      Added in the second para under the heading of methods in the first sentence.
   n) **How was risk of bias determined in the published studies?**
      Added in the second para under the heading of method
   o) **What were the inclusion and exclusion criteria?**
      The general criteria of inclusion and exclusion are now mentioned in the first paragraph under the heading of methods, however for individual intervention, we have described them in their respective section.
   p) **What constitutes the "Pre-conception" care?**
      Added in the first sentence of the first para under the heading of methods
   q) **Was "family planning" and "Pre-conception care" taken as synonymous?**
      No. I have now reviewed the term family planning which came in the text to see if it is giving this impression at all.
   r) **What about women of reproductive age with: Pre-pregnancy Respiratory disease (like cystic fibrosis); Endocrine problems (like polycystic ovarian syndrome) and women with Cancers (especially, hematologic malignancies Leukemia and lymphomas, breast cancer and cervical cancer)? Why were these conditions not studied as part of this review?**
      These conditions were reviewed but we did not find any evidence on these. We have now added them in the discussion to describe research gaps.

10) **Results:**
   i) **Provide a table for "characteristics of included studies" for each chronic disease. They can probably be included as web-tables**
      For the series, we can provide a link to the document where all the studies included in the whole series are detailed.
      https://globalmotherchildresearch.tghn.org/site_media/media/articles/Preconception_Report.pdf
   j) **Were there any studies that studied differential effect of Type 1 vs. type 2 diabetes on pregnancy outcomes?**
      Unfortunately not.
   k) **What pre-conception care was given in 21 studies included in meta-analysis for diabetes? Was it same in all the studies?**
      The preconception diabetes counseling in those studies were grouped as: 1) preconception counseling an glycemic control; 2) preconception counseling only; 3) glycemic control only. We have also run the meta-analyses accordingly as well. The results from those meta-analysis are presented in Figure 1 and figure 2.
   l) **It seems like that there was a lot of clinical heterogeneity among studies of pre-gestational diabetes in terms of study design, interventions and outcomes. Please comment on that.**
      The heterogeneity was moderate when we conducted the subgroup based on study design. However we have not showed them in the results. Study design: for congenital malformation: I^2 was 24% and P value of Chi^2 was 0.16. For perinatal mortality: I^2 was 0% and p value for chi^2 was 0.95 We have already presented the findings as per subgroup based on different interventions and it did not depict any significant heterogeneity.
   m) **First sentence on Section of Phenylketonuria is an exact copy of the first sentence of abstract of reference 66.**
      Sorry for that. We have now changed the sentence.

n) **There is a lot of redundant text in result's section that can be reduced by making tables for "Characteristics of included studies".**
We have removed the last paragraphs from each intervention and incorporated them in the discussion section

o) **It was noticed that authors tend to describe the complications of a particular medical condition rather than "pre-**conception care" especially towards the end of results section.
We have removed this section now.

p) Side effect of medications should be mentioned in their respective section.
We did not report side-effects with each of the drug as It will further increase the length of the paper. However, we have cautioned the risk of getting side-effects with medication use in the first paragraph under the heading of medication usage

11) Describe the limitations of the review.
12) What are the research gaps?

Added these two points in the discussion section.

Minor essential revisions:

5. **Abstract: Provide meaning of abbreviation used in Abstract: MNCH, CI, MD.**
Added
6. **Introduction: Give reference for the claims made in last paragraph of introduction section.**
References are not required here.
7. **Methods:**
   e) **What electronic databases were exactly searched? What was the search strategy?** Added
   f) **What "Standard quality criteria" was used to evaluate the quality of studies? Reference?**
   Added
   g) **How was Type 1 and type diabetes defined?** Added
   h) **What was the definition of Epilepsy?** Added

8. **Results:**
   f) **Flow diagram can further show the number of studies found for each aspect of the review (e.g. no of studies for diabetes out of 70.**
   We have described the number of studies for each section under their respective section. It would be too congested to add in the flow diagram.
   g) **Figure 1 and 2 are not visible in the document sent to me.**
   We have now zoomed the figures.
   h) **It is noticed that authors have referred results of previous reviews in result section on diabetes? Does this section include both discussion and results related to management of pre-gestational diabetes?**
   We have now addressed this issue and moved the reviews to the discussion section.
   i) **Section on Epilepsy: What does this sentence mean "Most women with epilepsy have no change in seizure frequency during pregnancy but about 15-33% have more seizures during pregnancy"? I think more seizures mean increase frequency???**
   Corrected "Most women with epilepsy have no change in seizure frequency during pregnancy but about 15-33% report increased episodes of seizures during pregnancy"
   j) **"Many studies looking at the effect of preconception counseling in women with chronic disorders versus healthy women have also looked into women with epilepsy but there comparison group was not relevant to this review, hence these studies were not included." What was that comparison group?**
   Removed this sentence it was not making sense.

Referee's comments to the authors– this sheet WILL be seen by the author(s) and published with the article

Title	**Preconception - Screening and management of chronic disease promoting psychological health**
Author(s)	Zohra S Lassi, Ayesha M Imam, Sohni V Dean, Zulfiqar A Bhutta
Referee's name	Khadija Humayun

When assessing the work, please consider the following points, where applicable:

1. Is the question posed by the authors new and well defined?
2. Are the methods appropriate and well described, and are sufficient details provided to replicate the work?
3. Are the data sound and well controlled?
4. Does the manuscript adhere to the relevant standards for reporting and data deposition?
5. Are the discussion and conclusions well balanced and adequately supported by the data?
6. Do the title and abstract accurately convey what has been found?
7. Is the writing acceptable?

Please make your report as constructive and detailed as possible in your comments so that authors have the opportunity to overcome any serious deficiencies that you find and please also divide your comments into the following categories:

- Major Compulsory Revisions (which the author must respond to before a decision on publication can be reached)
- Minor Essential Revisions (such as missing labels on figures, or the wrong use of a term, which the author can be trusted to correct)
- Discretionary Revisions (which are recommendations for improvement but which the author can choose to ignore)

Where possible please supply references to substantiate your comments.

When referring to the manuscript please provide specific page and paragraph citations where appropriate.

General comments:

Major compulsory revisions:

Minor essential revisions:
The Title and Abstract do not do not accurately convey the findings of the authors.

The title " Preconception - Screening and management of chronic disease promoting Psychological health" seems to tell that screening and management of chronic disease promotes psychological health, whereas the psychological illness has been seen as a form of chronic disease so it needs to be rephrased.
Rephrased as " Preconception care- Screening and management of chronic disease and promoting Psychological health"

The abstract does not summarize all the aspects the article deals with. In the abstract we get an idea about few things only
Discretionary revisions:

Continued:few issues only where as the review has much covered much more. Thus the abstract needs to be rewritten so that it is representative of the article.
Revisited and edited
